# Burden of intracerebral haemorrhage in Europe: forecasting incidence and mortality between 2019 and 2050

**DOI:** 10.1016/j.lanepe.2024.100842

**Published:** 2024-02-09

**Authors:** Hatem A. Wafa, Iain Marshall, Charles D.A. Wolfe, Wanqing Xie, Catherine O. Johnson, Roland Veltkamp, Yanzhong Wang, PRESTIGE-AF consortium, Roland Veltkamp, Kirsten H. Harvey, Eleni Korompoki, Lucio D’Anna, Omid Halse, Emily R. Harvey, Klemens Hügen, Uwe Malzahn, Sabine Ullmann, Carolin Schuhmann, Gabriele Putz Todd, Hannes Brinz, Cornelia Fiessler, Peter U. Heuschmann, Kirsten Haas, Viktoria Rücker, Christian Enzinger, Stefan Ropele, Daniela Pinter, Melanie Haidegger, Thomas Gattringer, Simon Fandler-Höfler, Charles D.A. Wolfe, Yanzhong Wang, Hatem A. Wafa, Joan Montaner, Elena Palà, Anna Penalba, Marcel Lamana Vallverdu, Daisy Guaman Pilco, Stéphanie Debette, Igor Sibon, Pauline Renou, Morgane Lachaize, Léa Milan, Nathalie Heyvang, Sylvain Ledure, Pascale Michel, Johanna Conhoc, Léa Donnadieu, Kelly Hyves, Valeria Caso, Maria Giulia Mosconi, Mara Graziani, Virginia Cancelloni, Laura Marchini, Bianca Emanuela Koehler, Peter Brønnum Nielsen, Torben Bjerregaard Larsen, Gregory Y.H. Lip, Solveigh Horstmann, Jan Purrucker, Peter Ringleb, Mariam Haffa, Sabrina Klein, Lenka Taylor, Torsten Hoppe-Tichy, Walter E. Haefeli, Hanna M. Seidling, Jürgen Burhenne, Kathrin I. Foerster, Viktoria Wurmbach, Claudia Marquart, Deirdre A. Lane, Gregory Y.H. Lip, Elena Ivany, Robyn Lotto

**Affiliations:** a School of Life Course and Population Health Sciences, King’s College London, London, UK; b National Institute for Health Research (NIHR) Collaboration for Leadership in Applied Health Research and Care (CLAHRC) South London, London, UK; c Institute of Health Metrics and Evaluation (IHME), University of Washington, Seattle, USA; d Department of Intelligent Medical Engineering, School of Biomedical Engineering, Anhui Medical University, Hefei, China; e Beth Israel Deaconess Medical Centre, Harvard Medical School, Harvard University, Boston, MA 02215, USA; f Department of Neurology, Alfried Krupp Krankenhaus Essen, Alfried-Krupp-Straße 21, Essen 45131, Germany; g Department of Brain Sciences, Imperial College London, London, UK

**Keywords:** Future, Stroke, Intracerebral haemorrhage, Epidemiology, Europe

## Abstract

**Background:**

Anticipating the burden of intracerebral haemorrhage is crucial for proactive management and building resilience against future health challenges. Prior forecasts are based on population demography and to a lesser extent epidemiological trends. This study aims to utilise selected modifiable risk factors and socio-demographic indicators to forecast the incidence and mortality of intracerebral haemorrhage in Europe between 2019 and 2050.

**Methods:**

Three intracerebral haemorrhage risk factors identified in the Global Burden of Diseases, Injuries, and Risk Factors study (GBD 2019)—high systolic blood pressure, high fasting plasma glucose, and high body mass index—were utilised to predict the risk-attributable fractions between 2019 and 2050. Disease burden not attributable to these risk factors was then forecasted using time series models (autoregressive integrated moving average [ARIMA]), incorporating the Socio-demographic Index (SDI) as an external predictor. The optimal parameters of ARIMA models were selected for each age-sex-country group based on the Akaike Information Criterion (AIC). Different health scenarios were constructed by extending the past 85th and 15th percentiles of annualised rates of change in risk factors and SDI across all location-years, stratified by age and sex groups. A decomposition analysis was performed to assess the relative contributions of population size, age composition, and intracerebral haemorrhage risk on the projected changes.

**Findings:**

Compared with observed figures in 2019, our analysis predicts an increase in the burden of intracerebral haemorrhage in Europe in 2050, with a marginal rise of 0.6% (95% uncertainty interval [UI], −7.4% to 9.6%) in incident cases and an 8.9% (−2.8% to 23.6%) increase in mortality, reaching 141.2 (120.6–166.5) thousand and 144.2 (122.9–172.2) thousand respectively. These projections may fluctuate depending on trajectories of the risk factors and SDI; worsened trends could result in increases of 16.7% (8.7%–25.3%) in incidence and 31.2% (17.7%–48%) in mortality, while better trajectories may lead to a 10% (16.4%–2.3%) decrease in intracerebral haemorrhage cases with stabilised mortality. Individuals aged ≥80 years are expected to contribute significantly to the burden, comprising 62.7% of the cases in 2050, up from 40% in 2019, and 72.5% of deaths, up from 50.5%. Country-wide variations were noted in the projected changes, with decreases in the standardised rates across all nations but varying crude rates. The largest relative reductions in counts for both incidence and mortality are expected in Latvia, Bulgaria, and Hungary—ranging from −38.2% to −32.4% and −37.3% to −30.2% respectively. In contrast, the greatest increases for both measures were forecasted in Ireland (45.7% and 74.4%), Luxembourg (45% and 70.7%), and Cyprus (44.5% and 74.2%). The modelled increase in the burden of intracerebral haemorrhage could largely be attributed to population ageing.

**Interpretation:**

This study provides a comprehensive forecast of intracerebral haemorrhage in Europe until 2050, presenting different trajectories. The potential increase in the number of people experiencing and dying from intracerebral haemorrhage could have profound implications for both caregiving responsibilities and associated costs. However, forecasts were divergent between different scenarios and among EU countries, signalling the pivotal role of public health initiatives in steering the trajectories.

**Funding:**

The European Union’s Horizon 2020 Research and Innovation Programme under grant agreement No. 754517. The 10.13039/501100000272National Institute for Health and Care Research (NIHR) under its Programme Grants for Applied Research (NIHR202339).

**Research in context**

**Evidence before this study**

A PubMed search was conducted without date or language restrictions up to 10 May 2023. We used terms such as “forecast”, “projection”, “stroke”, “cerebrovascular diseases”, “intracerebral haemorrhage”, “mortality”, “death”, “incidence”, and “burden” looking for matches in article titles and abstracts. From the resulting 169 records, we screened for original research articles offering forecasts for stroke burden. One of the largest and most recent studies forecasted a rise in death count from intracerebral haemorrhage by 6.6% between 2016 and 2040. Nonetheless, existing projections specific to Europe lacked consistent country-level estimates within a unified framework. Moreover, previous studies had limitations, such as assuming constant disease rates or neglecting the potential impact of trends in known risk factors associated with intracerebral haemorrhage. These limitations highlighted the necessity for a comprehensive set of forecasts that consider both incidence and mortality from intracerebral haemorrhage in Europe, while accounting for the intricate interplay between demographic patterns and risk factor exposures. The current study addresses these gaps and provides a more comprehensive outlook on the future burden of intracerebral haemorrhage in the European context.

**Added value of this study**

This study forecasted 0.6% and 8.9% rises in intracerebral haemorrhage cases and deaths, respectively, in Europe over the next three decades. The estimates were produced using a robust modelling framework that considered multiple risk factors, population demographics, and other independent drivers of socioeconomic changes. By leveraging data from the Global Burden of Diseases, Injuries, and Risk Factors Study, the study ensured the consistency of risk-outcome relationships with relevant cohort studies. The framework enabled the generation of reference forecasts and alternative scenarios, presenting a spectrum of potential trajectories based on different health trends. This approach goes beyond previous studies, offering valuable insights into the potential future burden of intracerebral haemorrhage and its implications for healthcare systems and policies at both regional and country levels.

**Implications of all the available evidence**

While age-standardised rates of intracerebral haemorrhage are predicted to maintain their downward trends, the absolute number of new cases and related deaths is expected to increase. The study highlights the need for proactive measures to address known modifiable risk factors, such as high systolic blood pressure, high fasting plasma glucose, and high body mass index, to mitigate the rising burden of intracerebral haemorrhage. Implementing effective preventive strategies might require development of novel approaches, especially among the elderly population who are more vulnerable and more likely to have co-existing diseases. Policymakers should consider tailoring interventions based on country-specific contexts, as the projected changes in intracerebral haemorrhage burden varied across European nations. Furthermore, the expected increase in the proportion of older adults underscores the importance of planning for the anticipated rise in healthcare demands and caregiving needs. Allocating sufficient resources and support for both affected individuals and their caregivers will be essential in addressing the challenges posed by intracerebral haemorrhage in the coming decades. Future research should continue to monitor epidemiological trends, risk factor prevalence, and disease mechanisms to refine projections and develop targeted interventions to combat the growing burden of intracerebral haemorrhage in Europe effectively.

## Introduction

Stroke has persistently ranked as a major cause of death and disability worldwide,^1^ with intracerebral haemorrhage constituting a significant proportion of stroke-related deaths (44%) despite contributing 28% of all stroke cases.^2^ Characterised by bleeding within the brain parenchyma, intracerebral haemorrhage leads to high fatality and disability rates, with survivors often experiencing severe neurological deficits.^3^^,^^4^ An expanding evidence from North America and Europe indicates a downward trend in stroke incidence and mortality rates,^5^, ^6^, ^7^, ^8^ with a pooled reduction in intracerebral haemorrhage incidence among high-income countries estimated at 37% between 1990 and 2010.^7^ Nevertheless, the ageing population and the reportedly rising prevalence of important risk factors such as obesity, diabetes, and hypertension,^9^ signal a likely increase in the burden of intracerebral haemorrhage over the coming years. Disease forecasts can facilitate the strategic allocation of necessary resources to address the growing demands of an ageing population, and, hence, help to mitigate the economic impacts on individuals and society. However, previous efforts to predict stroke occurrence and mortality have primarily focused on all stroke types combined, rather than distinct subtypes,^7^^,^^10^, ^11^, ^12^, ^13^, ^14^, ^15^ have relied on cross-sectional data and assumed static epidemiological trends,^13^, ^14^, ^15^ were based on expert consultation or simulation analyses,^16^ or applied basic regression techniques extrapolating disease patterns to population projections.^7^^,^^11^^,^^12^ While traditional models were limited in their ability to account for the dynamic and complex interplay of stroke risk factors, changing demographics, and advancements in medical care, the data needed to fit more sophisticated models are extensive and their availability remains limited.^10^^,^^17^^,^^18^

Foreman et al. projected an 8% worldwide rise in overall stroke fatalities between 2016 and 2040, with an expected 6.6% increase in deaths from intracerebral haemorrhage—growing from 2.84 million to 3.03 million.^17^ In contrast, a recent analysis based on the same data sources predicted a substantial decline of 22.3% in deaths caused by ischaemic stroke between 2020 and 2030.^19^ It is important to recognise that different methodologies can yield significantly varied estimates, underscoring the inherent limitations of forecasting techniques, since they are based solely on our knowledge of the past and present. This also emphasises the necessity of supplementing forecasts with alternative scenarios that explore a broader spectrum of plausible futures.^17^

In Europe, a recent initiative by the European Stroke Organisation has set an ambitious target to reduce the absolute number of strokes on the continent by 10% between 2018 and 2030.^20^ Despite the significance of this goal, the region currently lacks comprehensive projections for stroke types. Granular forecasts under various scenarios can offer valuable context, assisting healthcare officials in making the necessary adjustments to effectively work towards established targets. This study aims to produce refined forecasts of intracerebral haemorrhage incidence and mortality between 2019 and 2050 in Europe, overall, and by country. To generate these projections, a multifaceted forecasting framework, incorporating data on key risk factors, demographic transitions, and socioeconomic indices, was developed. In addition, we sought to demonstrate the possible trajectories in intracerebral haemorrhage outcomes under alternative assumptions.

## Methods

### Global Burden of Disease (GBD) data

The GBD is a comprehensive scientific endeavour aimed at measuring the relative extent of health loss attributed to diseases and injuries by age, sex, and location from 1990 to 2019 in 204 countries and territories. The conceptual and analytical structure for GBD and detailed methodologies have been reported elsewhere.^9^^,^^21^ The GBD protocol and data visualisation tools are also accessible online. In this section, the methods used to estimate the burden of intracerebral haemorrhage for GBD 2019 are briefly described, additional details are reported in the appendix (pp 5–10).

Stroke was defined in accordance with WHO clinical criteria^22^ as the rapid development of clinical signs, typically focal, indicating cerebral dysfunction lasting more than 24 h or leading to death. Intracerebral haemorrhage was specifically defined as a type of stroke characterised by a focal collection of blood in the brain not resulting from trauma. Intracerebral haemorrhage-related deaths were estimated using the Cause of Death Ensemble modelling (CODEm) framework with vital registration records as input data. CODEm incorporates geospatial relationships and information from covariates to guide modelled estimates. Estimates of intracerebral haemorrhage incidence were generated using the DisMod-MR 2.1 (Disease Model Meta-Regression) software,^23^ a Bayesian compartmental disease modelling tool used in GBD for non-fatal modelling. DisMod-MR leverages a combination of disease parameters, the epidemiological associations between these parameters, and geospatial data to produce estimates of intracerebral haemorrhage incidence. Inputs for the morbidity estimates included data on incidence, excess mortality and cause-specific mortality.

### Forecasting overview

We developed a modelling framework that builds upon the forecasting methodology of the GBD study, as initially introduced by Foreman and colleagues.^17^ A schematic diagram outlining the procedures followed for the projection of intracerebral haemorrhage burden is available in the appendix (Supplementary Fig. S1, pp 4). We trained our models on GBD data from the years 1990–2019 to create predictions of intracerebral haemorrhage incidence and mortality up to 2050 for 28 European nations, inclusive of the United Kingdom. Within the scope of this study, we refer to these 28 countries as “EU28”, “EU”, or “Europe”.

Rates of intracerebral haemorrhage incidence and mortality were modelled separately by country, age, and sex groups as a function of three independent components extended to 2050. The first component consisted of three key risk factors for intracerebral haemorrhage quantified in GBD 2019.^24^ The second component included time and a composite metric known as the Socio-demographic Index (SDI) combining income per person, educational attainment, and total fertility rate under the age of 25. The third component captured the unexplained secular trends over time. We specified the following relationship between the logarithm of each intracerebral haemorrhage outcome and the independent drivers:

$${\ln(m}_{casy})=\ln\left(S_{casy}\right)+\alpha_{cas}+\sum_{i=1}^{p}{AR}_{i}m_{{casy}_{-i}}^{e}+\sum_{j=1}^{q}{MA}_{j}\varepsilon_{{casy}_{-j}}^{e}+\beta \;{SDI}_{cy}\;+\;\epsilon_{casy}$$

where $S_{casy}$ is a country-age-sex-year-specific scalar combining the effects of GBD risk factors, $\alpha_{cas}$ is a constant term for the remaining time series, ${AR}_{i}$ is a coefficient for autoregressive association with i values ranging from 1 to p where p is the order of the autoregression, $m_{{casy}_{-i}}^{e}$ is the log-transformed risk-eliminated outcome at the previous year, ${MA}_{j}$ is a coefficient for moving average with j values ranging from 1 to q where q is the order of the moving average part. $\varepsilon_{{casy}_{-j}}^{e}$ is the error term from previous years, β is a global effect on SDI, and ϵ is a residual term estimated by use of a random walk ARIMA.

### The independent drivers forecasts

In this section, we outline a brief description of the procedures followed to predict the independent drivers (i.e., risk factors and SDI) needed to construct our reference forecasts. More details can be found elsewhere,^17^ and in the Supplementary appendix (pp 11–15). Our models incorporated three risk factors that have been quantified within the GBD 2019 framework and have well-established associations with intracerebral haemorrhage incidence and mortality: high systolic blood pressure, high fasting plasma glucose, and high body mass index (BMI).^9^^,^^24^ The risk-weighted prevalence of these risk factors was estimated using the summary exposure value (SEV), a univariate metric first introduced by GBD 2015 to account for different levels of exposure.^25^ We used SEVs to compute the annualised rates of change between 1990 and 2019, in the logit-transformed space, stratified by country, age, and sex groups. These values were projected into the future by calculating a weighted mean of prior changes using a recency weighting parameter (appendix, pp 12–13). Lastly, in order to capture the collective effects of the three GBD risk factors on intracerebral haemorrhage, we constructed risk factor scalars by converting the obtained SEVs (observed and forecasted) to population attributable fractions (PAFs). Joint PAFs were estimated after adjusting for the interplay of risk factors through one another using risk mediation factors provided in the GBD 2019 (appendix, pp 14–15).^9^ Finally, scalars were computed as:

$$S_{casy}=\frac{1}{1-{PAF}_{casy}}$$

where ${PAF}_{casy}$ is a country-age-sex-year-specific joint population attributable fraction of the three GBD risk factors.

In terms of the impact of population development, a single metric reflective of overall level of social and economic development was incorporated into our framework. The SDI is a summary measure combining three key dimensions closely related to health outcomes: income per capita (measured in gross domestic product adjusted for the purchasing power parity), educational attainment among individuals aged ≤15 years, and total fertility rate among women aged <25 years.^17^^,^^26^^,^^27^ These three factors are highly correlated and therefore SDI was included in our models rather than its individual factors to prevent variance inflation of parameter estimates. We projected SDI following the same technique described above by calculating weighted means of historical changes.

### Intracerebral haemorrhage forecasts

In both intracerebral haemorrhage incidence and mortality, we initially quantified risks that could not be attributed to specific risk factors. This was computed for each country, sex, and 5-year age group by dividing cohort-specific rate by the corresponding risk factor scalar. The resulting risk-eliminated outcomes were logit-transformed and subsequently projected to 2050 by fitting autoregressive integrated moving average (ARIMA) models using SDI as an external covariate. We used the Akaike Information Criterion (AIC) to determine the optimal order parameters of the ARIMA models (*p*,*d*,*q*). Unlike linear regression used in previous GBD forecasting papers,^28^ ARIMA models offered a more nuanced approach, capturing time series dynamics while accounting for additional relevant information. Moreover, in a unique aspect of our analyses, we considered the potential delayed effect of SDI on intracerebral haemorrhage outcomes by testing the 10-year out-of-sample predictive accuracy with various lagged time intervals (1–5 years). The optimal lag identified was then applied to the full series and used for forecasting.

The residuals derived from these models, representing the unexplained variation in intracerebral haemorrhage outcomes, were further analysed and forecasted by fitting a random walk model [ARIMA_(0,1,0)_]. These residuals were added to our final predictions for intracerebral haemorrhage—calculated by multiplying the projected risk-eliminated outcomes by the forecasted risk factor scalar. Ultimately, future counts of intracerebral haemorrhage cases and deaths were calculated as the product of the obtained risk estimates and GBD population forecasts.^29^ For standardisation, we used the 2013 European Standard Population to calculate adjusted risks of intracerebral haemorrhage incidence and mortality.

### Worse and better health scenarios and uncertainty

To explore alternative trajectories, we defined both worse and better scenarios using a percentile-based approach of the independent drivers. Specifically, we identified the 15th and 85th percentiles of the weighted annualised rates of change observed across all European countries in the past for SDIs and risk factors. Calculated for each age and sex cohort as appropriate, these percentiles served as benchmarks for constructing hypothetical future trends, illustrating the potential outcomes for intracerebral haemorrhage incidence and mortality under optimistic/pessimistic levels of change in all independent drivers. We implemented specific adjustment when the reference scenario fell outside the defined alternative trajectories. In such instances, we shifted the divergent scenario to align with the reference forecast. This adjustment aimed to prevent countries with rates of change outside the 15th and 85th percentiles from regressing/improving beyond these values in the alternative assumption.

In our estimation process, we implemented bootstrapping to address uncertainty. At each stage, we performed 1000 random sampling. This technique enabled us to account for variability and uncertainty originating from different components of the analysis. Uncertainty intervals (UIs) were defined as the 25th and 975th values of the ordered sample draws. By using these percentiles, we effectively captured the range of uncertainty in our projections.

### Decomposition analysis

We utilised methodologies devised by Das Gupta in order to assess the relative influence of population growth, age demographics, and risk factors on intracerebral haemorrhage trends.^30^ These techniques mathematically isolate the standardised effect of each multiplicative factor, enabling us to quantify the individual contribution of each element to the changes in intracerebral haemorrhage burden.

### Framework validation and performance assessment

To evaluate the overall validity of our methods we held the last 10 years of observed data (2010–2019) and trained the entire framework on data from 1990 to 2009. The procedure was also applied to the algorithms used to forecast input data (i.e., the independent drivers). We then compared the out-of-sample forecasts for the test period with the actual observed data using the root-mean-squared error (RMSE). The overall performance and other detailed results by different cohorts of age, sex, and country can be found on pages 34–50 of the appendix. The findings demonstrate that our models outperformed the widely used demographic approach, the Lee-Carter method,^31^ and the average annualised rate of change extrapolation method. Finally, we re-fitted our models using the complete datasets from 1990 to 2019 to produce projections up to 2050.

All analyses were performed using the statistical software R version 4.2.2. Ethics approval was not required since all data used in this study are de-identified and publicly available.

### Role of the funding source

The study funders had no role in study design, data collection, data analysis, data interpretation, or the writing of the manuscript. The projected is funded by European Union’s Horizon 2020 Research and Innovation Programme under grant agreement No. 754517. In addition, this project is funded by the National Institute for Health and Care Research (NIHR) under its Programme Grants for Applied Research (NIHR202339) and is supported by the NIHR Applied Research Collaboration (ARC) South London at King’s College Hospital NHS Foundation Trust. The views expressed are those of the authors and not necessarily those of the NIHR or the Department of Health and Social Care.

## Results

The European burden of intracerebral haemorrhage is expected to increase between 2019 and 2050 in both absolute and relative terms. However, controlling for the anticipated changes in population age and sex composition showed a potentially declining trajectory.

**Fig. 1:**
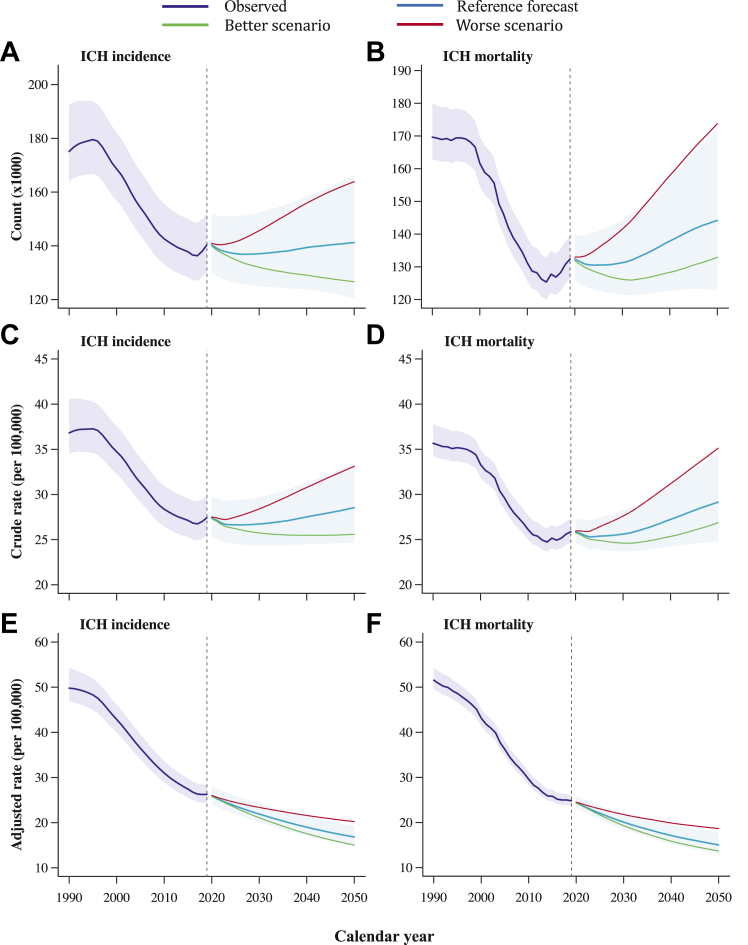
Intracerebral haemorrhage incidence and mortality over time in Europe, with projections up to 2050. ICH indicates intracerebral haemorrhage.

In Table 1, we present the observed number of new intracerebral haemorrhage cases in 2019 and their corresponding 2050 forecasts under different health scenarios (reference, better, and worse) for the 28 countries of the EU. The table also highlights the percentage change in both count and age-standardised rates between 2019 and the 2050 reference forecast. Additional insights into historical changes over the past three decades can be found in Supplementary Table S2 in the appendix (pp 24–38). Overall, we estimated a marginal increase of 0.6% (95% UI, −7.4% to 9.6%) in the number of new intracerebral haemorrhage cases by 2050. Should EU countries experience worse health trends in socioeconomic and intracerebral haemorrhage risk factors, a 16.7% (95% UI, 8.7%–25.3%) increase was forecasted with the total number of new sufferers potentially reaching 163.9 (95% UI, 141.6–190.3) thousand in 2050 (Table 1, Fig. 1). Conversely, if countries followed the better health trajectory, a 10% (95% UI, 16.4%–2.3%) decline in intracerebral haemorrhage cases was estimated. Regarding mortality, our projections indicate that 144.2 (95% UI, 122.9–172.2) thousand people will die from intracerebral haemorrhage in 2050, representing an 8.9% increase compared to 2019. This contrasts with the preceding 22% decline observed since 1990 in the region (Fig. 1, Table 2). In the worse health scenario, a 31.2% (95% UI, 17.7%–48%) increase in deaths was predicted whereas status quo was forecasted under the better health scenario (0.4%; 95% UI, −10.7% to 13.5%).

**Table 1:** Number of intracerebral haemorrhage cases in 2019 and 2050 and percentage change in counts and age-standardised rates by country.

	Number of new intracerebral haemorrhage cases				Percentage change (2050 vs 2019)	
	2019	2050 forecast	2050 better scenario	2050 worse scenario	Count	Age-standardised rates
EU28	140,388	141,202	126,606	163,861	0.6 (−7.4 to 9.6)	−35.9 (−32 to −38.8)
Austria	1,919	1,737	1,325	1,786	−9.5 (−17.3 to −1.5)	−49.2 (−45.2 to −51.7)
Belgium	2,798	3,425	3,102	3,856	22.4 (12.8–34.3)	−28.7 (−23.2 to −32.7)
Bulgaria	6,261	4,233	3,874	4,821	−32.4 (−36 to −28.5)	−32.9 (−28.7 to −36.1)
Croatia	1,536	1,214	1,001	1,329	−21 (−26.1 to −13.5)	−37.4 (−33 to −40.5)
Cyprus	263	379	360	425	44.1 (34.9–53.4)	−32.2 (−27.4 to −35.5)
Czechia	2,687	2,219	1,948	2,540	−17.4 (−23.8 to −10.4)	−41.3 (−37.3 to −44.2)
Denmark	1,221	1,286	1,162	1,419	5.3 (−4.2 to 15.2)	−38.7 (−33.7 to −42.5)
Estonia	316	239	237	309	−24.4 (−28.6 to −18.6)	−40.9 (−37.5 to −43.1)
Finland	1,644	1,784	1,696	2,047	8.5 (−2 to 22.1)	−32.1 (−26.9 to −36.1)
France	13,955	14,719	12,968	16,654	5.5 (−4.3 to 18.2)	−37.1 (−32.8 to −40.5)
Germany	19,016	18,020	16,994	21,604	−5.2 (−13.4 to 3.6)	−40.1 (−35.7 to −43.4)
Greece	5,732	6,319	5,920	7,259	10.2 (−0.9 to 22.9)	−30.6 (−24.8 to −34.8)
Hungary	3,347	2,426	1,943	3,165	−27.5 (−30.9 to −23.6)	−41.9 (−38.9 to −43.9)
Ireland	737	1,075	1,009	1,319	45.7 (36.3–57.3)	−29.2 (−24.8 to −32.6)
Italy	16,557	17,812	13,862	18,105	7.6 (0.5–15.3)	−30.2 (−25.5 to −34)
Latvia	801	495	482	599	−38.2 (−41.5 to −33.2)	−38.4 (−34.4 to −40.4)
Lithuania	979	783	703	835	−20 (−24.1 to −15.1)	−22.8 (−19.4 to −25.9)
Luxembourg	105	153	136	180	45 (32.8–60.7)	−39.6 (−34 to −43.3)
Malta	114	131	109	150	14.8 (7.6–20.5)	−40 (−36.2 to −43.3)
Netherlands	3,721	4,623	4,517	5,548	24.2 (13.4–36.4)	−29.4 (−23.7 to −33.5)
Poland	11,144	10,878	9,051	11,472	−2.4 (−7.9 to 5.2)	−33.2 (−29.4 to −36.2)
Portugal	3,623	3,564	2,923	3,744	−1.6 (−8.1 to 5.3)	−34.1 (−30.1 to −37.7)
Romania	12,287	9,750	9,657	12,246	−20.6 (−25.7 to −13.8)	−40.7 (−36.5 to −43.5)
Slovakia	1,514	1,514	1,235	1,628	0 (−4.8 to 5.6)	−35.5 (−31.9 to −38.3)
Slovenia	512	580	495	672	13.3 (4.1–25.3)	−34.8 (−29.9 to −38.4)
Spain	11,184	10,759	11,196	16,649	−3.8 (−11.7 to 3.4)	−46 (−41.9 to −49)
Sweden	2,802	3,379	3,210	3,848	20.6 (12.5–29)	−27.2 (−22 to −31)
United Kingdom	13,603	17,692	15,470	19,649	30.1 (19.7–41.2)	−24.2 (−18.9 to −27.6)

Figures between brackets are the 95% UIs. Further details including estimates with the 95% UI are included in Supplementary Table S3 in the Supplementary appendix.

**Table 2:** Number of deaths from intracerebral haemorrhage in 2019 and 2050 and percentage change in counts and age-standardised rates by country.

	Number of deaths due to intracerebral haemorrhage				Percentage change (2050 vs 2019)	
	2019	2050 forecast	2050 better scenario	2050 worse scenario	Count	Age-standardised rates
EU28	132,404	144,170	132,939	173,756	8.9 (−2.8 to 23.6)	−39.4 (−31.7 to −45.3)
Austria	1,280	1,546	1,157	1,711	20.8 (7–37.3)	−43 (−35.4 to −48.8)
Belgium	2,845	3,474	3,521	4,379	22.1 (8.9–36.8)	−33.7 (−25.4 to −40)
Bulgaria	7,608	5,075	5,106	6,423	−33.3 (−38.6 to −26.9)	−37.5 (−31 to −42.9)
Croatia	1,607	1,156	1,035	1,478	−28.1 (−35.2 to −19.2)	−46.2 (−38.8 to −51.2)
Cyprus	216	376	357	420	74.2 (57–93.3)	−28.6 (−19.3 to −36)
Czechia	1,931	1,498	1,480	2,078	−22.5 (−30 to −13.5)	−50.5 (−44.1 to −55.4)
Denmark	1,178	1,489	1,203	1,491	26.5 (10.8–46.5)	−35.3 (−26.1 to −42.2)
Estonia	201	198	136	181	−1.4 (−10.9 to 11.1)	−34.8 (−26.4 to −40.4)
Finland	1,152	1,621	1,290	1,562	40.7 (23.9–61.2)	−19.1 (−8.5 to −27.3)
France	12,107	13,771	10,690	14,607	13.7 (−1.5 to 35)	−42.6 (−34.6 to −48.5)
Germany	15,532	17,239	15,210	18,383	11 (−1.2 to 24)	−34.4 (−26.6 to −40.8)
Greece	6,476	7,475	6,695	9,061	15.4 (2.4–32.3)	−32.2 (−23.4 to −38.7)
Hungary	2,528	1,764	1,243	2,293	−30.2 (−37.2 to −19.5)	−51.4 (−45.3 to −56)
Ireland	492	858	713	923	74.4 (54.9–95.1)	−31.2 (−22.5 to −38.7)
Italy	18,307	23,811	24,192	29,203	30.1 (13.7–49.8)	−33.1 (−21.9 to −41)
Latvia	672	421	317	542	−37.3 (−43.7 to −28.7)	−47.2 (−40.7 to −51.6)
Lithuania	663	531	508	595	−19.9 (−27 to −11.6)	−32 (−24.9 to −37.9)
Luxembourg	103	176	147	197	70.7 (50–94.6)	−37.9 (−28.9 to −45)
Malta	86	92	74	96	6.4 (−3.8 to 17.5)	−51 (−45.3 to −55.5)
Netherlands	3,144	4,558	4,020	5,052	45 (28.3–64.4)	−28.4 (−18.7 to −35.5)
Poland	9,893	10,791	9,013	11,800	9.1 (−1.2 to 21.7)	−35.2 (−28 to −40.9)
Portugal	4,236	4,109	2,968	3,993	−3 (−13.2 to 9.2)	−41.9 (−34.3 to −47.9)
Romania	12,917	9,078	9,919	12,637	−29.7 (−35.6 to −22.8)	−50.6 (−45.5 to −54.7)
Slovakia	1,308	1,245	1,134	1,610	−4.8 (−13.1 to 4.4)	−46.8 (−41.5 to −51.4)
Slovenia	460	492	501	656	7 (−4 to 20.3)	−44.3 (−36.3 to −50.3)
Spain	11,170	11,869	10,520	18,630	6.3 (−5.8 to 20)	−49.7 (−42.5 to −54.8)
Sweden	1,920	2,119	2,584	2,947	10.3 (−2.2 to 24.7)	−41.1 (−33 to −47.3)
United Kingdom	12,394	17,362	17,190	20,817	40.1 (24.7–58.6)	−27.4 (−18.3 to −34.5)

Figures between brackets are the 95% UIs. Further details including estimates with the 95% UI are included in Supplementary Table S4 in the Supplementary appendix.

**Fig. 2:**
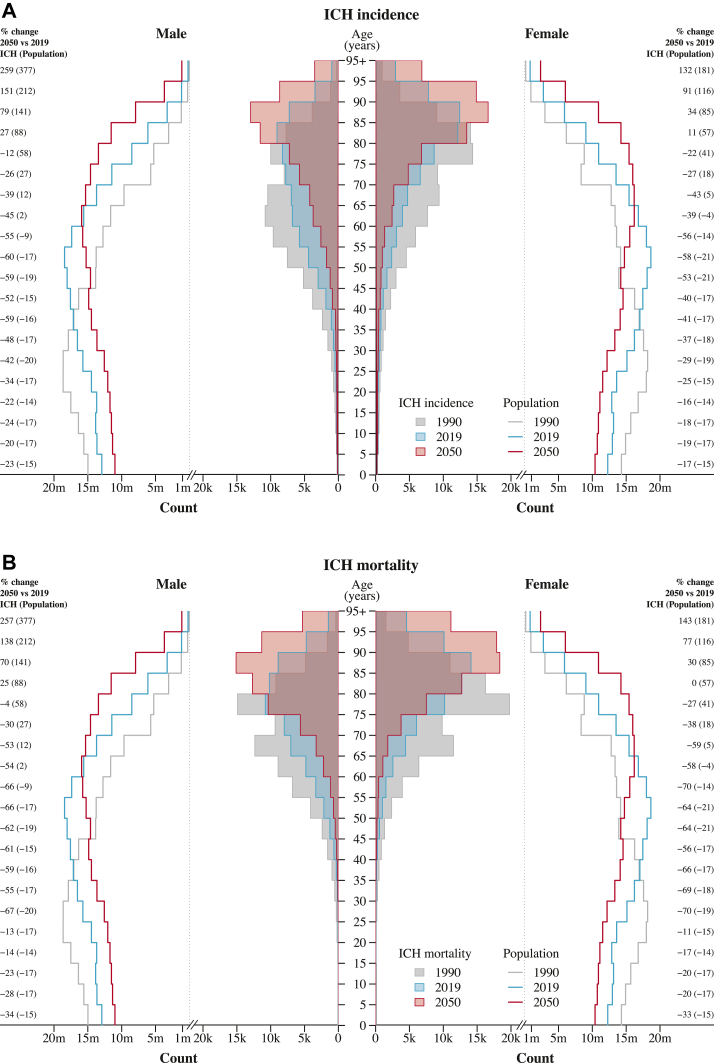
Intracerebral haemorrhage incidence and mortality by age and sex groups in 1990, 2019, and 2050 reference forecast, along with European population structures. ICH indicates intracerebral haemorrhage.

The absolute burden of intracerebral haemorrhage in Europe was observed to be slightly higher in females than males, with significant disparity widening with age (Fig. 2). Generally, for every five-year increment in age until 80 years old, there was an approximate one-third rise in the number of intracerebral haemorrhage cases, while mortality nearly doubled. Such pattern was expected to continue in the future, with individuals aged 80 years and above forecasted to contribute 62.7% and 72.5% of all incidence and deaths from intracerebral haemorrhage, respectively, rising from the 40% and 50.5% levels seen in 2019. In the year 2050, the estimated incidence rate in males was predicted to be 10.7 per 100,000 individuals aged 40–59 years, 35.3 among those aged 60–79, and 149.9 among those aged 80 years and above. The corresponding European projections in females were estimated at 6.4, 26.1, and 153.8 cases per 100,000. On the other hand, the forecasted rates of death per 100,000 males were 4.3 among those aged 40–59 years, 36.2 among those aged 60–79, and 181.7 among those aged 80 years and above. For females, the projected mortality rates were 2, 22.1, and 178.5 per 100,000 (Supplementary Table S1 in the appendix, pp 16).

**Fig. 3:**
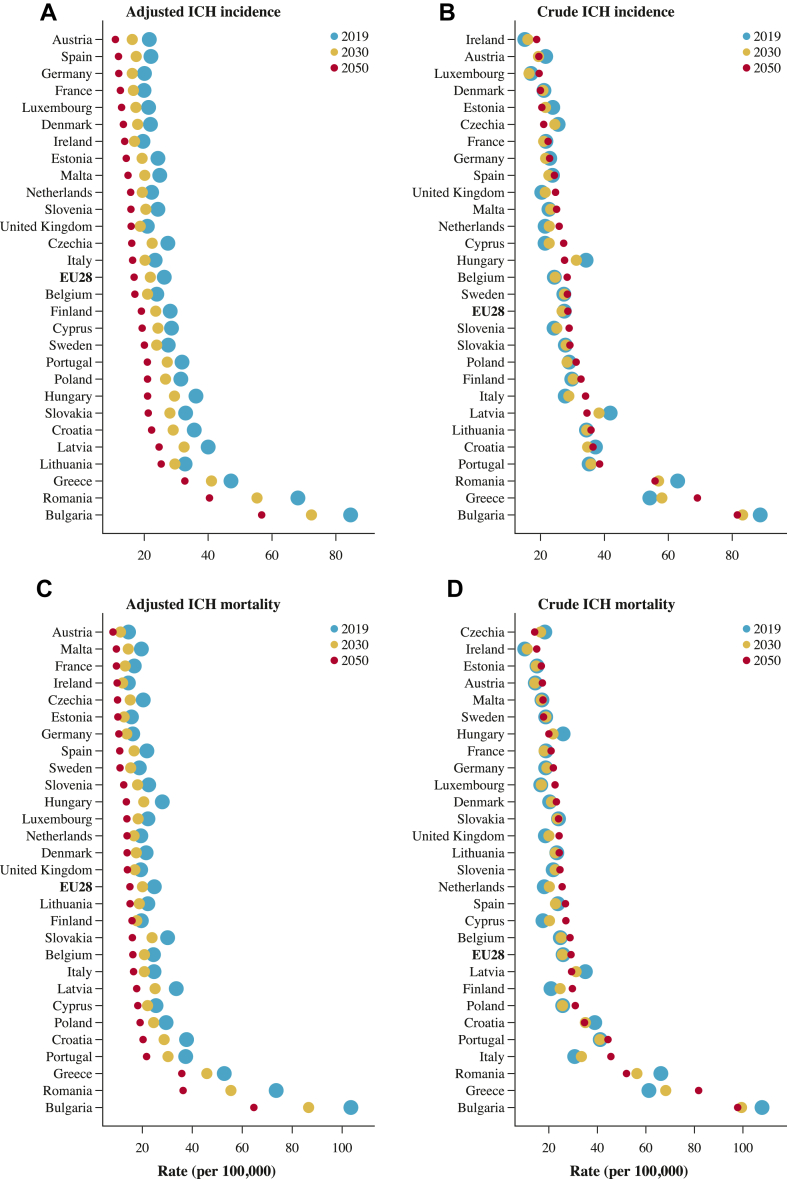
Observed and projected changes in intracerebral haemorrhage incidence and mortality rates between 2019 and 2050 in Europe. The size of the dots does not convey specific information; it is utilised to illustrate the direction of the time series and to enhance clarity in cases of overlap. Countries arranged in ascending order according to the 2050 forecasts. ICH indicates intracerebral haemorrhage.

**Fig. 4:**
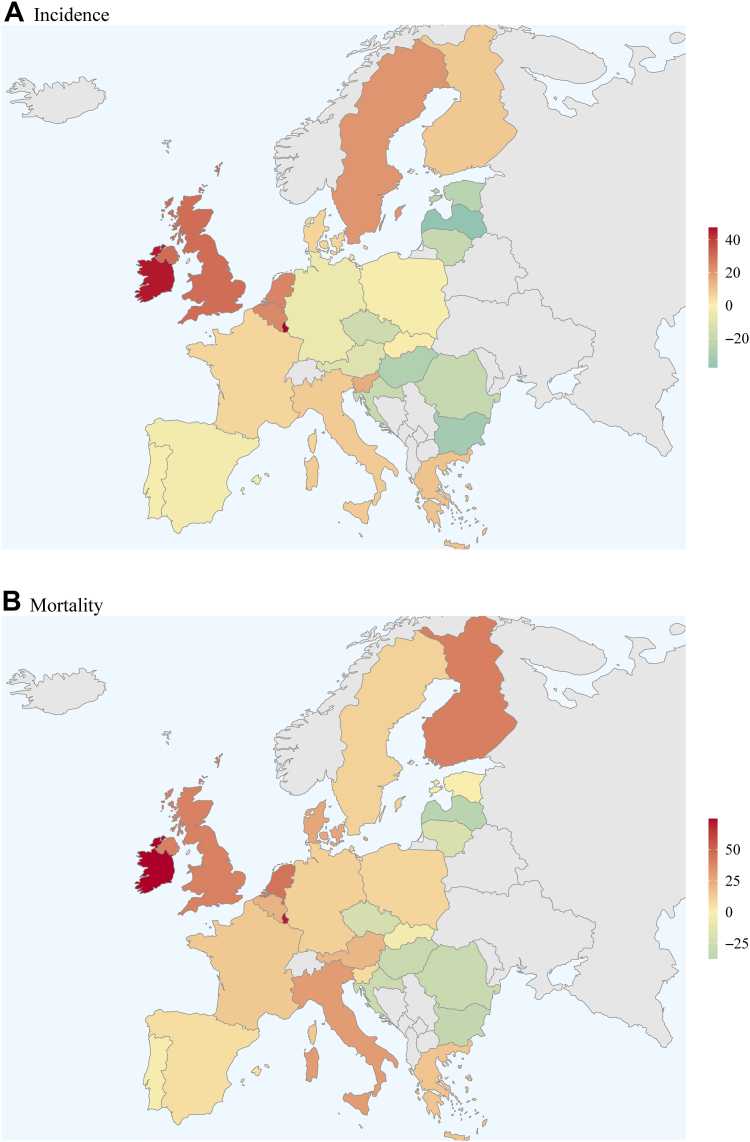
Percentage change in numbers of intracerebral haemorrhage incidence and mortality in 2050 vs 2019.

**Fig. 5:**
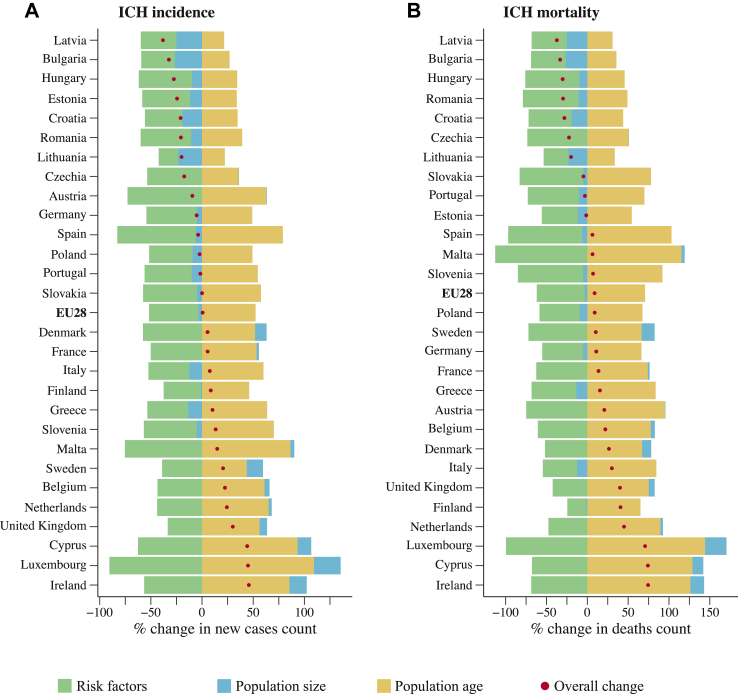
Decomposition of percentage change in the number of intracerebral haemorrhage cases and deaths between 2019 and 2050 in Europe. Countries arranged in ascending order according to their overall forecasted change. ICH indicates intracerebral haemorrhage.

Upon analysing further patterns by country, notable variations in the projected intracerebral haemorrhage figures were evident. Austria was estimated to have the lowest age-standardised incidence and mortality rates of 11 cases and 8.3 deaths per 100,000 in 2050, respectively, whereas the greatest rates of 56.8 and 64.7 were expected in Bulgaria. Although there were decreases in the standardised rates for every country, ranging from approximately one-fifth to half, the majority of EU countries will either experience a rise or a slight decline in crude rates and count figures (Fig. 3, Fig. 4, Fig. 5). In age-standardised terms for intracerebral haemorrhage incidence, the most significant declines between 2050 and 2019 were expected in Austria (−49.2%), Spain (−46%), and Hungary (−41.9%). Conversely, Lithuania (−22.8%), the UK (−24.2%), and Sweden (−27.2%) were predicted to witness the smallest decreases. Likewise, adjusted mortality rates showed significant drops, with Hungary, Malta, and Romania experiencing declines as high as −51.4%, −51%, and −50.6%, compared to less significant changes of 19.1%, −27.4%, and −28.4% in Finland, the UK, and the Netherlands.

Trends of intracerebral haemorrhage incidence and mortality will largely be shaped by the unfolding patterns in demography and key independent drivers. Age-wise, the proportion of children and young adults (aged 0–59) is expected to decrease in the EU, while the proportion of older adults (aged 60 and above) is projected to increase (Supplementary Table S2 in appendix, pp 22). Particularly, the proportion of people living to the age 80 years or more is expected to more than double from 5.8% in 2019 to 11.8% in 2050. In addition, with the ongoing and forecasted improvements in SDI, the current adverse trends in metabolic and behavioural risk factors, including, hypertension, diabetes, and high BMI, are expected to persist until 2050 (Supplementary Figs. S2–S7 in the appendix, pp 17–23). Even under the favourable assumption of health changes, the prevalence of high fasting blood glucose and high BMI is set to continue its upward trajectory. Despite these adverse trends, the net effect of intracerebral haemorrhage risks will continue to exert a positive influence on the overall trends (Fig. 5). In fact, improvement in intracerebral haemorrhage risk in general will offset a significant proportion of the increased burden attributable to population ageing. However, population ageing is expected to remain the predominant factor by 2050.

## Discussion

This study presents a comprehensive set of forecasts for intracerebral haemorrhage incidence and mortality over the next three decades in Europe. These projections are complemented by alternative scenarios illustrating the spectrum of potential trajectories based on different health trends. On a positive note, our findings suggest that age-standardised rates are predicted to maintain their downward trends, albeit at a slower pace, indicating progress in mitigating individual risks. However, the EU will still likely witness a rise in both intracerebral haemorrhage cases and related deaths. This mounting burden will fall on a population marked by a decreasing proportion of individuals in the working age class, and is therefore poised to have profound implications for healthcare systems and societal responsibilities.^32^^,^^33^

The results from our study present a complex picture of the anticipated future burden of intracerebral haemorrhage in Europe, highlighting a multifaceted scenario that is primarily influenced by evolving demographic patterns, the effects of key risk factors, and socioeconomic conditions. Historically, the impact of improving risk factors and their management has outweighed the effects of population ageing, thus leading to a decline in the overall burden of intracerebral haemorrhage. However, projections for the next three decades suggest a notable shift in this dynamic. Anticipated improvement due to better socioeconomic circumstances and other factors will be opposed by predicted rises in the prevalence of hypertension, diabetes, and obesity—partly diminishing the beneficial effects of these positive interventions. As a result, the concurrent effects of population ageing, especially the rising proportion of individuals aged 80 years and above, will surpass the positive effects of health improvements leading to stabilisation or even reversal in intracerebral haemorrhage absolute burden in the majority of EU countries. The expected surges in new cases and deaths from intracerebral haemorrhage in Europe emphasises the persistent and upcoming challenge for healthcare systems. Our results indicate that the burden of intracerebral haemorrhage will continue to be a pressing issue, particularly among older adults, considering the ageing European demographic. The impact of such an increase could be substantial, stretching healthcare resources and placing a significant burden on the economy. Hence, policymakers should consider proactive and innovative strategies to address the potential increase in healthcare demands associated with intracerebral haemorrhage. Increasing investment in research, especially evidence-based practices for enhancing stroke prevention, holds significant potential to mitigate the costs associated with the rising demand. In the UK, for instance, stroke experts estimated that allocating £10 million to each priority research area could yield savings of approximately £4 billion for stroke prevention and £1.3 billion for treatment research by 2035.^34^^,^^35^ However, the disparity observed across countries calls for tailored strategies taking into account specific country contexts and the unique demographic, socioeconomic, and health profiles of their populations.

In parallel with Foreman et al.’s estimate of a 6.6% increase in global intracerebral haemorrhage deaths by 2040,^17^ our analysis showed a similar yet slightly higher increase of 8.7% in Europe for the same timeframe. Both studies employed similar projection methodologies and this difference is likely due to the relatively higher life expectancy in Europe than the global average. On the other hand, comparing our country-specific estimates of intracerebral haemorrhage mortality with other studies showed varied results. Our forecast in the United Kingdom, for instance, is comparable to the 16.4% decline in deaths count estimated by Kunst and colleagues between 2005 and 2030—the corresponding change estimated from our models was −21%.^11^ Notably, Kunst et al. analysed combined data for all stroke types, not exclusively intracerebral haemorrhage, thus our estimates may not necessarily align with their findings. In the Netherlands, Kunst’s analysis estimated a 31.2% increase in stroke deaths, in contrast to our forecasted 11.8% increase, while in Sweden, their projected 10% rise exceeds our modest prediction of 0.14%. On a different note, it is noteworthy to mention that in countries such as the Netherlands, where the number of deaths from intracerebral haemorrhage was predicted to exceed the number of new cases, several factors could contribute to this phenomenon. These include changes in disease duration and improvement in diagnosis and treatment practices. However, it is crucial to recognise that mortality rates could be more influenced by disease prevalence rather than incidence. In other words, deaths can occur from a larger pool of survivors in addition to incident cases. And with the ongoing advancement in treatment options, more people are likely to survive their first intracerebral haemorrhage, leading to an increasing prevalence and thus larger mortality.

When put in the context of the Stroke Action Plan for Europe (SAP-E) by the European Stroke Organisation,^20^ our research findings portray a diverse pattern across individual countries within the EU. The SAP-E targets a 10% reduction in the number of all new strokes by 2030, potentially achievable through a significant decrease in strokes of ischaemic nature. However, haemorrhagic strokes may not meet this target. Our analysis suggests a 0.6% rise in incident cases by 2050, or at best, a 10% decline. Nevertheless, by extrapolating from our data it becomes apparent that some countries are on a promising trajectory toward this goal while others face considerable challenges. For instance, Bulgaria was predicted to experience a drop of 16.2% in new cases by 2030, and Austria was expected to observe an 11.8% reduction, positioning them favourably to achieve the SAP-E target. Conversely, countries like Ireland and the Netherlands were forecasted to have concerning 17.7% and 11.9% increases, respectively, in new intracerebral haemorrhage cases. Furthermore, the UK with a projected 30.1% increase by 2050 indicates a need for strategic intervention. Importantly, however, nations with considerable predicted improvements currently exhibit higher rates of intracerebral haemorrhage incidence and mortality, in age-standardised terms. Besides, these countries have a larger prevalence of risk factors. As a result, the anticipated improvements in such countries may be primarily driven by their increased capacity for improvement, stemming from their worse current profiles, rather than more effective management practices. In summary, our study emphasises that while progress is being made, the goal of reducing intracerebral haemorrhage incidence by 10% by 2030 will require concerted efforts and a better understanding of risk factor management in the context of frailty and multimorbidity, particularly in countries where incidence rates are projected to rise.

While our study provides valuable insights, it is important to acknowledge several limitations. Our analysis relied on data from the GBD 2019 study, which synthesised estimates of intracerebral haemorrhage incidence and mortality from a diverse set of input sources. It is important to note that variations may exist between GBD estimates (1990–2019) and other national and subnational figures due to differences in methodologies and data sources. Nevertheless, the primary emphasis of this study was to project the expected changes in the future relative to the latest observed figures, and GBD data offered valid estimates within a unified framework that were highly comparable among countries and across time. More importantly, however, as inherent to any forecasting study, our estimates were based on specific assumptions about future trajectories. The covariates inputted in our models, including risk factors and populations demography, were themselves forecasts which introduces additional element of uncertainty. And while they have proven robust in out-of-sample performance—demonstrated when our models were trained on data from 1990 to 2009 and tested against the period from 2010 to 2019—the reliability of such performance in future projections is not guaranteed. Nonetheless, we have supplemented our forecasts by considering two additional scenarios—illustrating the extent to which our estimates were sensitive to changes in input variables and providing insights into what might be achievable under different future conditions. On a different note, our forecasting methodology assumed that the examined risk factors were causally related to the incidence and mortality of intracerebral haemorrhage. Although we utilised established data sources and adjusted for various covariates, the possibility of non-causal associations or unaccounted for confounding variables cannot be entirely ruled out. Further research is needed to establish stronger causal relationships between risk factors and intracerebral haemorrhage outcomes. Additionally, the lack of temporal trend data for intracerebral haemorrhage incidence and mortality posed a challenge in incorporating observed time trends into our forecasted projections. Considering the evolving diagnostic procedures and changes in healthcare practices over time, allowing observed time trends to influence future projections could lead to spurious forecasts. Therefore, our forecasts were primarily based on expected trends in the prevalence of risk factors, population size, and composition. To enhance the accuracy and reliability of future projections, further investment in high-quality epidemiological studies is necessary. These studies should focus on estimating trends in intracerebral haemorrhage measures and geographical variations over time. Additionally, expanding the scope of risk factors included in the analysis and incorporating more comprehensive data and the effects of risk factor managements would enable a more thorough understanding of the associations between risk factors and control measures and intracerebral haemorrhage outcomes. Despite these limitations, our study successfully incorporated significant risk factors, such as hypertension, obesity, fasting plasma glucose, and SDI, which are expected to undergo substantial changes in the future. While the exclusion of certain risk factors was inevitable due to methodological constraints, our projections remain valuable in highlighting the potential impact of these selected risk factors on future outcomes of intracerebral haemorrhage in Europe. Nonetheless, future endeavours should aim to include a wider range of risk factors within established frameworks, allowing for a more comprehensive analysis without relying solely on ecological associations between risk exposure prevalence and intracerebral haemorrhage outcomes.

In conclusion, despite the considerable progress that has been made in Europe in reducing intracerebral haemorrhage risk and associated mortality, the projected trends suggest an increasing number of individuals afflicted by and dying from this condition. In fact, without sustained efforts to enhance the prevalence and control of risk factors and to advance treatment options, the number of intracerebral haemorrhage cases and related deaths could revert back to levels comparable to those observed in the 1990s. It is vital that such efforts are informed by a better understanding of risk factor management in the context of increased frailty and comorbidity of an ageing population.

## Contributors

Conceptualisation: Hatem A. Wafa, Yanzhong Wang. Data curation: Hatem A. Wafa. Formal analysis: Hatem A. Wafa. Funding acquisition: Roland Veltkamp, Iain Marshal, Charles D. A. Wolfe. Methodology: Hatem A. Wafa, Yanzhong Wang, Wanqing Xie. Project administration: Hatem A. Wafa. Supervision: Yanzhong Wang. Validation: Hatem A. Wafa. Visualisation: Hatem A. Wafa. Writing—original draft: Hatem A. Wafa. Writing—review & editing: Yanzhong Wang, Iain Marshal, Charles D. A. Wolfe, Wanqing Xie, Catherine O Johnson, Roland Veltkamp, PRESTIGE-AF consortium. Hatem A. Wafa and Yanzhong Wang had access to and verified the underlying data. Yanzhong Wang is responsible for the decision to submit the manuscript.

## Data sharing statement

The data used in this study can be obtained from the Global Health Data Exchange at https://ghdx.healthdata.org/gbd-2019.

## Declaration of interests

Professor Roland Veltkamp reports consulting fees from Bayer, Astra Zeneca, Bristol Myers Squibb, and Pfizer; payment for lectures from Astra Zeneca; ownership of stocks in Novartis and Bayer; leadership roles in the World Stroke Organisation Board of Directors and Research committee; institutional support outside the submitted work from Medtronic, Daiichi Sankyo, Bristol Myers Squibb, and Pfizer; and participation in Bayer’s Data Safety Monitoring/Advisory Boards. Other authors reported no conflict of interests.

## Appendix ASupplementary data

Suppementary appendix
