# Supplementary material for: Burden of intracerebral haemorrhage in Europe: forecasting incidence and mortality between 2019 and 2050
Source: Lancet Reg Health Eur. 2024 Feb 9;38:100842. doi: 10.1016/j.lanepe.2024.100842 (PMC10867656; doi:10.1016/j.lanepe.2024.100842)
Supplement: Suppementary appendix [file mmc1.pdf]

Suppementary appendix

Supplement to: Burden of intracerebral haemorrhage in Europe: forecasting incidence and mortality between 2019 and 2050

Hatem A Wafa, Iain Marshall, Charles D. A. Wolfe, Wanqing Xie, Catherine O Johnson, Roland Veltkamp, Yanzhong Wang, on behalf of the PRESTIGE-AF consortium.

Table of Contents

Summary of GBD methods for estimation of ICH & risk factors 5

ICH input data 5

Modelling strategy for ICH death 7

Definitions of risk factors 8

Relative risks estimation 9

Summary exposure value estimation methods 11

Summary exposure value projection methods 12

Population-attributable fraction and scalar estimation methods 14

Framework performance – out-of-sample predictive validity 43

References 51

List of Tables

Table S1: Incidence and mortality rates of ICH by age and sex groups in Europe. 16

Table S2: Population demography and ICH incidence and mortality by EU country, in 1990, 2019, and 2050 reference forecast. 24

Table S3: Number of ICH cases in 2019 and 2050 and percentage change in counts and age-standardised rates by country...39

Table S4: Number of deaths from ICH in 2019 and 2050 and percentage change in counts and age-standardised rates by country. 41

List of Figures

Figure S1: Forecasting flowchart. 4

Figure S2: Independent drivers forecasts by country and overall..18

Figure S3: The independent drivers in Europe over time (1990-2050). 19

Figure S4: Sociodemographic index in selected European countries (1990-2050). 20

Figure S5: High systolic blood pressure in selected European countries (1990-2050). 21

Figure S6: High fasting plasma glucose in selected European countries (1990-2050). 22

Figure S7: High body mass index in selected European countries (1990-2050). 23

Figure S8: Overall out-of-sample predictions for ICH incidence and mortality in Europe.....43

Figure S9: Out-of-sample predictions for ICH incidence and mortality in Europe by sex groups...44

Figure S10: Out-of-sample predictions for ICH incidence in Europe by age groups.............46

Figure S11: Out-of-sample predictions for ICH mortality in Europe by age groups. 48

Figure S12: Out-of-sample predictions for ICH incidence in selected European countries....49

Figure S13: Out-of-sample predictions for ICH mortality in selected European countries. ...50

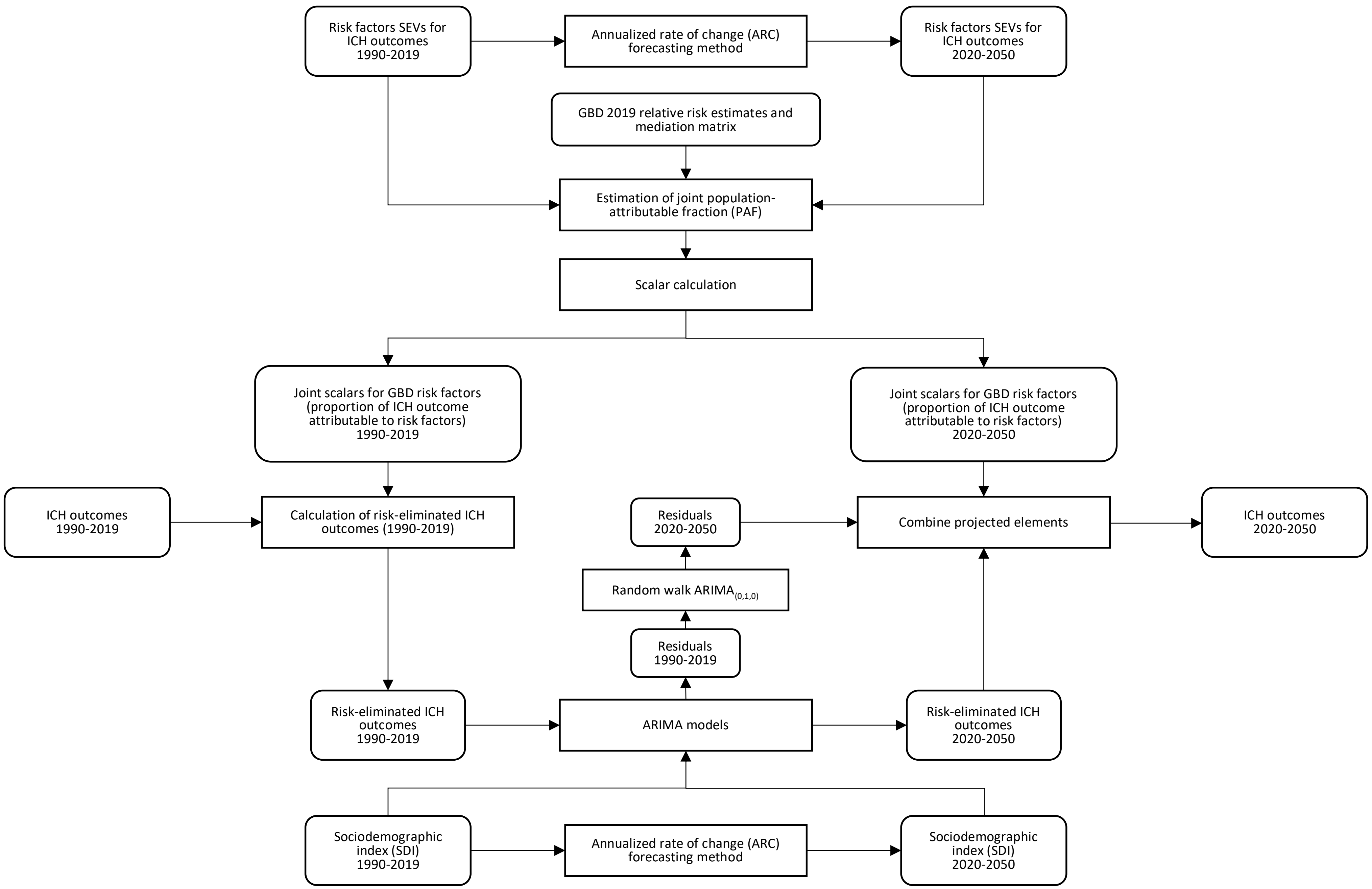

Figure S1: Forecasting flowchart.

Summary of GBD methods for estimation of ICH & risk factors

(Adapted from "Global, regional, and national burden of stroke and its risk factors, 1990–2019: a systematic analysis for the Global Burden of Disease Study 2019" by Feigin et al., The Lancet Neurology. 2021 Oct 1;20(10):795–820.)[1]

The results from the GBD 2019 study are accessible through the Global Health Data Exchange (GHDx), which is the most comprehensive repository of health-related data, including surveys, censuses, vital statistics, and more. The GHDx offers an interactive data downloading tool that allows users to access the GBD 2019 results. The GBD 2019 results are measured in terabytes, reflecting the extensive amount of data available. The latest version of the data download tool can be accessed at this link: https://ghdx.healthdata.org/. This tool provides core summary results for GBD 2019, encompassing various health metrics such as deaths, years of life lost (YLLs), years lived with disability (YLDs), disability-adjusted life-years (DALYs), prevalence, incidence, and rate of change. Additionally, the GHDx includes data on causes, risks, cause-risk attribution, aetiologies, and impairments.

While some data can be viewed online, larger datasets require downloading. Users may need to provide their email address, as the files will be prepared and a download location will be sent to them. The GBD 2019 online data visualizations are available at https://www.healthdata.org/gbd/data-visualizations, offering results for all GBD health metrics. These platforms provide valuable resources for researchers, policymakers, and other stakeholders to access and explore the comprehensive GBD 2019 data, enabling informed decision-making and further analysis in the field of global health.

Intracerebral haemorrhage input data

The GBD 2019 study utilizes a wide range of data sources to synthesize information on morbidity, mortality, and attributable risk for 204 countries and territories. These data sources include surveys, censuses, vital statistics, and other health-related data. To access the specific data input sources used in the GBD study, an interactive citation tool is provided through the GHDx. The Data Input Sources Tool in GHDx, accessible at http://ghdx.healthdata.org/gbd-2019/data-input-sources, allows users to view and access GHDx records for the input sources. It also enables users to export a comma-separated value (CSV) file containing metadata, citations, and information about where the data were used in the GBD study. This tool helps

researchers and stakeholders access the specific sources used in the study and explore the associated metadata.

The CoD (Causes of Death) database used in the GBD 2019 study includes various types of data sources, such as vital registration, verbal autopsy, cancer registries, police records, sibling history, surveillance, survey/census data, and minimally invasive tissue sample (MITS) diagnoses. While complete vital registration systems provide comprehensive cause of death information, not all countries have such systems in place. For countries with incomplete vital registration systems, other data types may be used to supplement the vital statistics for causes of death. Overall, the GBD 2019 study ensures transparency and accessibility of the data sources used through the interactive citation tool and provides a comprehensive range of information on morbidity, mortality, and attributable risk worldwide.

To improve the representation of population-level disease incidence for intracerebral hemorrhage (ICH) in the GBD 2019 study, several adjustments were made to the data prior to analysis using the DisMod-MR modeling tool. These adjustments were performed using the MR-BRT (Bayesian regularized model with temporal smoothing) modeling tool. The purpose of these adjustments was to enhance the accuracy and reliability of the estimates. Specifically, the study accounted for various study-specific factors when adjusting the data. These factors included determining whether the data originated from hospital sources and whether the data included both first-ever and recurrent cases of ischemic stroke. Adjustments were made to ensure that the analysis appropriately accounted for these factors, resulting in more robust and accurate estimates of disease incidence. Additionally, the methods for redistributing deaths attributed to unspecified stroke, indicated by ICD-10 codes I62 and I64, were updated to assign them to the three stroke subtypes modeled in the GBD study. This refinement allowed for a more detailed and accurate representation of stroke subtypes.[2]

Verbal autopsy data and vital registration data were utilized in modeling cerebrovascular disease, which includes stroke. In the analysis, deaths reported in verbal autopsy reports for cerebrovascular disease among individuals under 20 years of age were reassigned to the parent category of cardiovascular disease for both sexes. This adjustment ensured consistency and improved the accuracy of the analysis.

During the analysis, non-representative subnational verbal autopsy data points were identified as outliers and excluded from the analysis. Additionally, ICD8 data points that exhibited inconsistency with the rest of the data and created implausible time trends were identified as

outliers. These outliers were excluded from the analysis, ensuring that the remaining data points were more representative and aligned with the overall trends. Similarly, data points from sources that were deemed implausibly low in all age groups and those causing the regional estimates to be unrealistically high were also identified and treated as outliers.

	Incidence	Mortality
**First-ever acute haemorrhagic stroke**		
Central Europe	3	1
Eastern Europe	5	2
Western Europe	29	17
**Chronic haemorrhagic stroke**		
Central Europe	0	1
Eastern Europe	0	0
Western Europe	0	7

Counts of data used by measure of hemorrahge strokes for GBD 2019 in Europe.

Modelling strategy for ICH death

CODEm (Cause of Death Ensemble modeling) is an analytical tool used in the estimation of trends in causes of death.[3] It employs a comprehensive approach that involves exploring a wide range of possible models to estimate these trends. The covariate selection algorithm within CODEm identifies numerous plausible combinations of covariates. These combinations are then evaluated using four different model classes, which include mixed effects linear models and spatial-temporal Gaussian Process Regression models for cause fractions and death rates. This approach allows for a thorough exploration of potential models that can capture the complex dynamics of causes of death.

To assess the performance of the models, out-of-sample predictive validity is employed. This means that models are tested on data that were not used in the model fitting process, providing an independent assessment of their predictive capabilities. The models with the best out-of-sample predictive performance are selected and combined into an ensemble model. The ensemble model leverages the strengths of each individual model and outperforms any single component model in terms of metrics such as root mean square error, the frequency of correctly predicting temporal trends, and achieving 95% coverage of the prediction interval.

In modeling deaths from ICH for the GBD 2019 study, a standard CODEm (Cause of Death Ensemble modeling) approach was employed. Several adjustments were made to the dietary covariates used in the analysis.

Adjusted dietary covariates for the consumption of fruits, omega-3 fatty acids, vegetables, nuts and seeds, and polyunsaturated fatty acids were replaced with summary exposure value scalars for a diet low in each of these factors. The direction of these dietary covariates was changed from -1 to 1, as it was assumed a priori that low levels of intake of these dietary factors are associated with an increased risk of mortality from intracerebral hemorrhage. Furthermore, the dietary covariate for whole grains (kcal/capita, adjusted) and the social demographic index covariate were dropped from the analysis, as exploratory analyses indicated that these covariates were not predictive of mortality risk from intracerebral hemorrhage. Additionally, adjustments were made to other covariates. The direction of the covariate for alcohol was changed from 0 to 1, reflecting the a priori hypothesis about the association between alcohol consumption and mortality risk from intracerebral hemorrhage. The level and direction of the cholesterol covariate were also modified, changing from 1 to 3 and from 0 to -1, respectively, to reflect the mixed and inconclusive evidence regarding cholesterol levels and the risk of intracerebral hemorrhage. The level of the trans fatty acid covariate was also changed from 1 to 3, aligning with the expected importance of this risk factor on mortality from intracerebral hemorrhage.

It is important to note that apart from these covariate adjustments, there were no other substantive changes from the approach used in GBD 2017 in the modeling of deaths from intracerebral hemorrhage for the GBD 2019 study.

Definitions of risk factors

A BMI greater than 23.0 kg/m^2 is regarded as high, as is fasting plasma glucose exceeding 5.4 mmol/L. High systolic blood pressure refers to measurements greater than 110-115 mmHg. Definitions of theoretical minimum risk exposure levels have been detailed elsewhere.[4,5]

Relative risks estimation

In the GBD 2019 study, a systematic approach was used to gather data on relative risks (RRs) for various risk factors and their association with ICH outcomes. Below are the key steps and methods involved:

1. Data Collection: Primary studies, including randomized controlled trials (RCTs), cohort studies, pooled cohort studies, and case-control studies, were collected for the risk-outcome pairs included in GBD 2019. Meta-analyses and systematic reviews were also utilized to gather RRs.
2. RR Estimation: RRs were estimated based on the available data from the collected studies. The RRs were determined as a function of exposure to the risk factor, and different RRs were incorporated for mortality and morbidity outcomes.
3. Confounding Control: The RRs used in the analysis controlled for confounding factors but not for factors along the causal pathway between exposure and outcome.
4. Conversion of Exposure Categories: For risks estimated from continuous exposure distributions, where studies reported effect sizes by categories, the categories were converted to RRs per unit increase in exposure assuming a linear increase in the log of the RR and exposure.
5. Modelling RRs: For selected continuous risk factors, RRs were modelled using meta-regression with cubic splines. The MR-BRT program, a set of wrappers customized for global health problems (https://github.com/zhengp0/limetr),[6] was used for this purpose. The risk factors modelled with MR-BRT included dietary risk factors, low physical activity, kidney dysfunction, and air pollution.
6. Systematic Literature Reviews: Systematic literature reviews were conducted for 18 risks, while for other risk factors, data were primarily sourced from household surveys and other non-published sources. Data from household surveys, such as Demographic

and Health Surveys, Multiple Indicator Cluster Surveys, and Living Standards Measurement Surveys, were systematically screened and included in the analysis.

7. Data Management: Information on systematic reviews and data sources was managed using the Research Electronic Data Capture (REDCap) electronic data capture tools.[7]

The data sources, citations, and additional details can be accessed through the GHDx and the associated web tools.

For more comprehensive details and specific risk factors, it is recommended to refer to the original GBD 2019 study documentation and resources (http://ghdx.healthdata.org).

Summary exposure value estimation methods

(Adapted from "Forecasting life expectancy, years of life lost, and all-cause and cause-specific mortality for 250 causes of death: reference and alternative scenarios for 2016–40 for 195 countries and territories" by Foreman et al., The Lancet 2018; 392: 2052–90.)[8]

Our models incorporated three risk factors that have been quantified within the GBD 2019 framework and have well-established associations with ICH incidence and mortality: high systolic blood pressure, high fasting plasma glucose, and high body mass index (BMI).[5,9] The risk-weighted prevalence of these risk factors were estimated using the summary exposure value (SEV), a univariate metric first introduced by GBD 2015 to account for different levels of exposure.[10] SEVs range from 0 to 1, where a value of 0 indicates no risk and a value of 1 suggests that the entire population is at maximum risk.

$$SEV_{ro} = \frac{\int_{l}^{L} p_1 RR_1 dl - RR_{min}}{(RR_{max} - 1)RR_{min}}$$

where l denotes a category of exposure, as in low, medium, or high. If we set $RR_{min} = 1$

$$SEV_{ro} = \frac{\int_{l}^{L} p_1 RR_1 dl - 1}{RR_{max} - 1}$$

Where RR_{max} is the relative risk at the highest level of exposure in theory or observed globally. Stated in terms of a population attributable fraction (PAF), the SEV is

$$SEV_{ro} = \frac{PAF_{rc}}{(1 - PAF_{ro})(RR_{max} - 1)}$$

In order to only have one SEV per risk factor, we averaged it across the causes which are affected by each risk factor:

$$SEV_r = \frac{1}{N(o)} \sum_{o} SEV_{ro}$$

For our forecasting work we computed a SEV for each risk factor, because SEVs have the advantage of being a univariate variable that is simple to forecast while still capturing the complex relationships between risk factors and causes of death. These SEVs are then used to generate population attributable fractions (PAFs) and cause-specific scalars, using the methods detailed later in this material.

Summary exposure value projection methods

(Adapted from "Forecasting life expectancy, years of life lost, and all-cause and cause-specific mortality for 250 causes of death: reference and alternative scenarios for 2016–40 for 195 countries and territories" by Foreman et al., The Lancet 2018; 392: 2052–90.)[8]

For each risk factor (r), we calculated the annual change in the logit of the SEV for every country (c), age (a), sex (s), and past year (y = 1991, ... ,2019).

$$d_{c,a,s,y,r} = \text{logit}(SEV)_{c,a,s,y,r} - \text{logit}(SEV)_{c,a,s,y\text{-}1,r}$$

We then computed the annualized rate of change for each country, age, and sex by calculating the weighted mean of the first difference over time, where the weights w_{y} are determined by a recency-weighting parameter ω, and scaled to sum to 1.

$$\delta_{c,a,s,r} = \text{mean}(d_{c,a,s,r,\mathrm{Y}}, w_{r,\mathrm{y}})$$

$$w_{r,\mathrm{y}} = (\mathrm{y} - \mathrm{y}_{initial} + 1)^{\omega_r}$$

In order to select weighting parameters ω, we used data from just 1990 to 2009 to project each risk factor to 2019 using values of ω ranging, in increments of 0.25, from 0 to 10. We then calculated the root-mean-square error ($RMSE$) out-of-sample. We took the reciprocal of the RMSE values for the ω values, and defined the resulting values as the probabilities of selecting their corresponding ω values. So the probability of selecting ω_i would be:

$$P(\omega_i) = \frac{\frac{1}{RMSE_{\omega_i}}}{\sum_{\omega}^{\vec{\omega}} \frac{1}{RMSE_{\omega}}}$$

Where $\vec{\omega}$ is the list of all tested ω values. We used these probabilities to produce a multinomial distribution of ω values. We forecasted SEVs for each risk factor using a random sample of values from this distribution. This approach helped us avoid choosing values for ω that were based on inconsequential differences in the RMSE, placing more weight on the earlier part of the time series by biasing the distribution of ω values toward zero, and add uncertainty to the SEV forecasts. Finally, to make our reference scenario for each SEV, we simply applied the weighted mean first differences into the future, starting from 2019.

$$SEV_{c,a,s,y,r} = expit\left(logit\left(SEV_{c,a,s,2019,r}\right) + \delta_{c,a,s,r} \times (y - 2019)\right)$$

Forecasts that fell outside the 15th and 85th percentiles of change in logit SEVs were replaced with the nearest values of these percentiles. This approach is designed to prevent extreme forecasts based on outlier trends, therefore enhancing the reliability of overall projections.[8,11,12]

To capture the collective effects of the three GBD risk factors on ICH, we constructed risk factor scalars specific for each country, age, sex, and year (past and forecasted), following the same approach described in detail in previous GBD and detailed below.

Population-attributable fraction and scalar estimation methods

(Adapted from “Forecasting life expectancy, years of life lost, and all-cause and cause-specific mortality for 250 causes of death: reference and alternative scenarios for 2016–40 for 195 countries and territories” by Foreman et al., The Lancet 2018; 392: 2052–90.)

To project future Population Attributable Fractions (PAFs) for a specific risk factor (r), we transformed the predicted SEVs into PAFs as follows.

$$\widehat{PAF}_{rocay} = 1 - \frac{1}{\widehat{SEV}_{rcay} \times (RR_{ro}^{max} - 1) + 1}$$

$\widehat{PAF}$ estimates depend on $\widehat{SEV}$ which is not ICH-specific. As a result, we anticipate a bias in the logit-transformed space, where exposures are modeled. We try to correct for this bias by aligning our estimated values with the GBD data for the year 2019. This alignment is achieved by initially referring to a PAF that is directly computed from exposure and cause-specific relative risks available in the GBD:

$$PAF_{rocay} = \frac{\sum_x^X p_{xrcay} \times RR_{xro} - 1}{\sum_x^X p_{xrcay} \times RR_{xro}}$$

where x corresponds to the different exposure levels of the risk factor. This is followed by calculating the correction factor CF via comparing (in logit space) the GBD PAF to the $\widehat{SEV}$-derived estimated $\widehat{PAF}$ in the reference year 2019:

$$CF_{roca} = logit(PAF_{roca2019}) - logit(\widehat{PAF}_{roca2019})$$

This correction factor is necessary because the SEV is summarized across all of the causes of death related to that risk factor. If there are different patterns of relative risk by exposure level for different causes of death for the same risk factor, there is some information loss attributable to this dimensionality reduction. Since that correction factor is relatively stable over time, we can simply add it to each year in the forecast to approximate the cause-risk-specific $\widehat{PAF}$ accounting for these different relative risk curves.

We applied the correction factor to the estimated $\widehat{PAF}$ to come up with an adjusted estimated PAF^*:

$$PAF_{rocay}^* = expit(logit(\widehat{PAF}_{rocay}) + CF_{roca})$$

To properly estimate the joint PAF of all risks, one must take into account of how one risk factor is mediated through other risk factors. The fraction of one risk that is mediated through another is called Mediation Factor (MF). Using risk mediation factors provided in the GBD 2019, we computed the joint (adjusted) PAF of all risks for an outcome:

$$PAF_{ocay} = 1 - \prod_{r}^{R}(1 - PAF^{*}_{rocay} \times \prod_{s}^{S}(1 - MF_{rso}))$$

Where $s \in S$ denotes the risks that impact the outcome o via r, and $r \in R$ are all the risks associated with outcome o. Since PAF is the ratio of risk-attributable ICH outcome to total ICH outcome, we can relate total rate of ICH outcome to underlying rates.

$$PAF = \frac{ICH\ outcome\ attributable\ to\ GBD\ risk\ factors}{total\ ICH\ outcome}$$

&

$$Total\ ICH\ outcome = ICH\ outcome\ attributable\ to\ GBD\ risks + outcome\ not\ attributable\ to\ GBD\ risks$$

therefore,

$$Total\ ICH\ outcome = outcome\ not\ attributable\ to\ GBD\ risks\ \times \frac{1}{1-PAF}$$

Finally, we generated a risk factor scalar, corresponding to the ratio of total ICH outcome to underlying rate.

$$Scalar = \frac{1}{1 - PAF}$$

Table S1: Incidence and mortality rates of ICH by age and sex groups in Europe.

	Males		Females	
	2019	2050	2019	2050
**Incidence rate**				
Children (0 to 9)	1.40	1.32	2.29	2.24
Adolescents (10-19)	1.97	1.79	2.84	2.78
Young adults (20-39)	4.13	2.54	3.87	3.08
Middle-aged adults (40-59)	20.96	10.65	11.40	6.44
Older adults (60-79)	60.25	35.27	42.10	26.14
Elderly (>=80)	190.03	149.94	189.05	153.83
**Mortality rate**				
Children (0 to 9)	0.04	0.03	0.03	0.01
Adolescents (10-19)	0.08	0.06	0.07	0.04
Young adults (20-39)	0.91	0.47	0.45	0.20
Middle-aged adults (40-59)	10.34	4.30	4.67	1.95
Older adults (60-79)	61.94	36.15	40.90	22.08
Elderly (>=80)	231.89	181.74	222.04	178.49

Rates presented for 2050 are the reference forecasts.

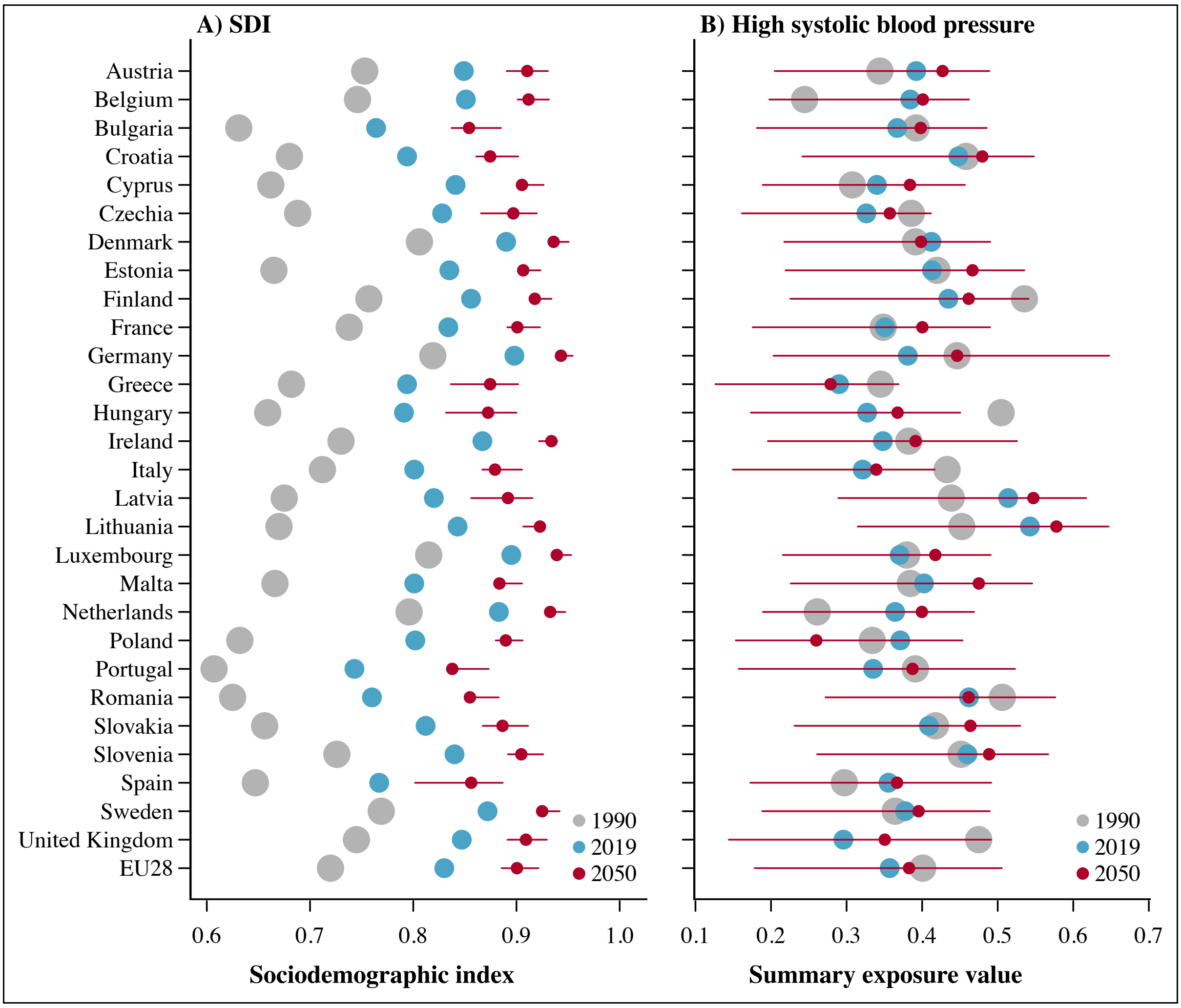
A) SDI
B) High systolic blood pressure
Austria
Belgium
Bulgaria
Croatia
Cyprus
Czechia
Denmark
Estonia
Finland
France
Germany
Greece
Hungary
Ireland
Italy
Latvia
Lithuania
Luxembourg
Malta
Netherlands
Poland
Portugal
Romania
Slovakia
Slovenia
Spain
Sweden
United Kingdom
EU28
1990
2019
2050
0.6 0.7 0.8 0.9 1.0
Sociodemographic index
0.1 0.2 0.3 0.4 0.5 0.6 0.7
Summary exposure value

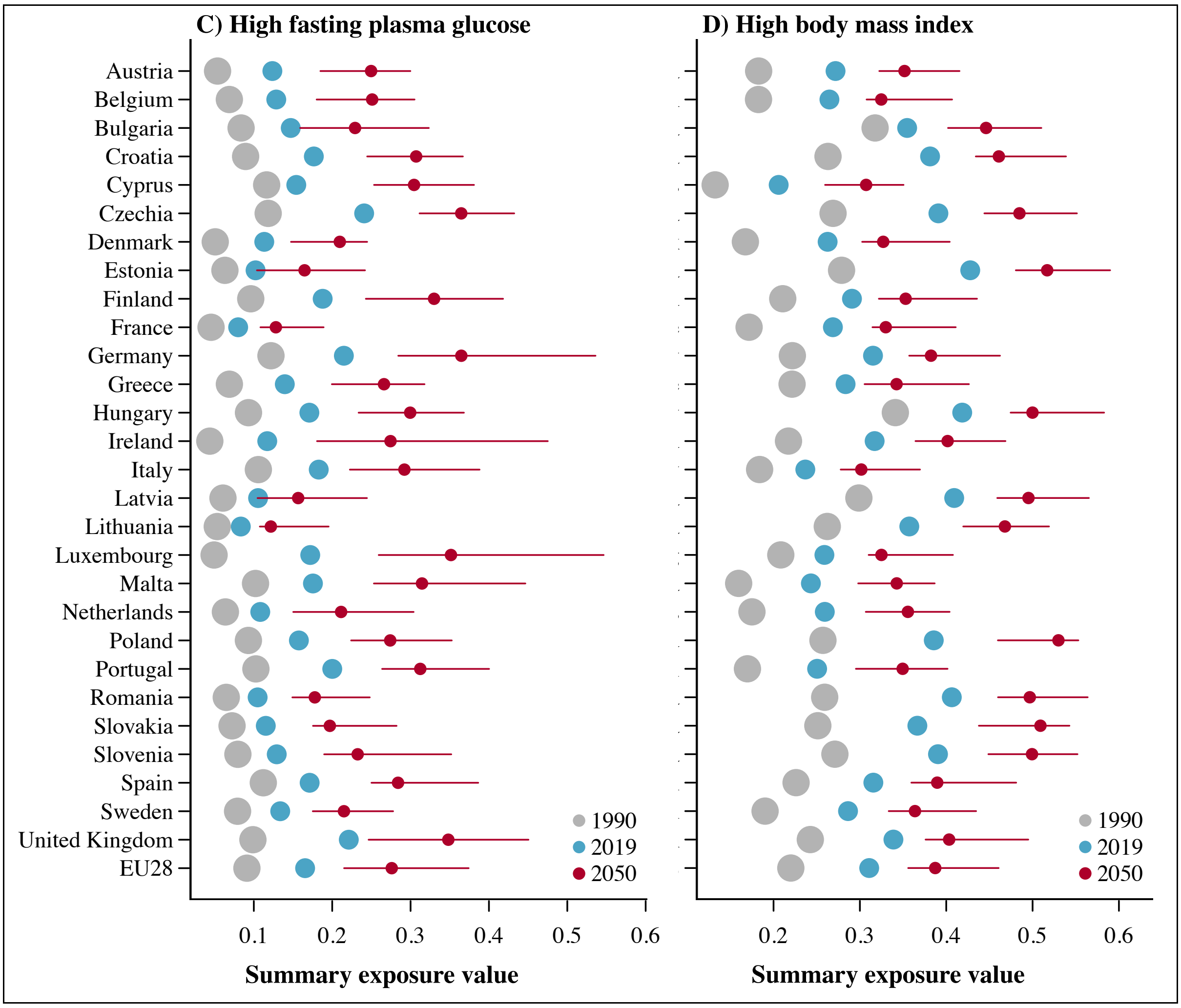

Figure S2: Independent drivers forecasts by country and overall.

Lines around the year 2050 indicate the range of worse and better scenarios.

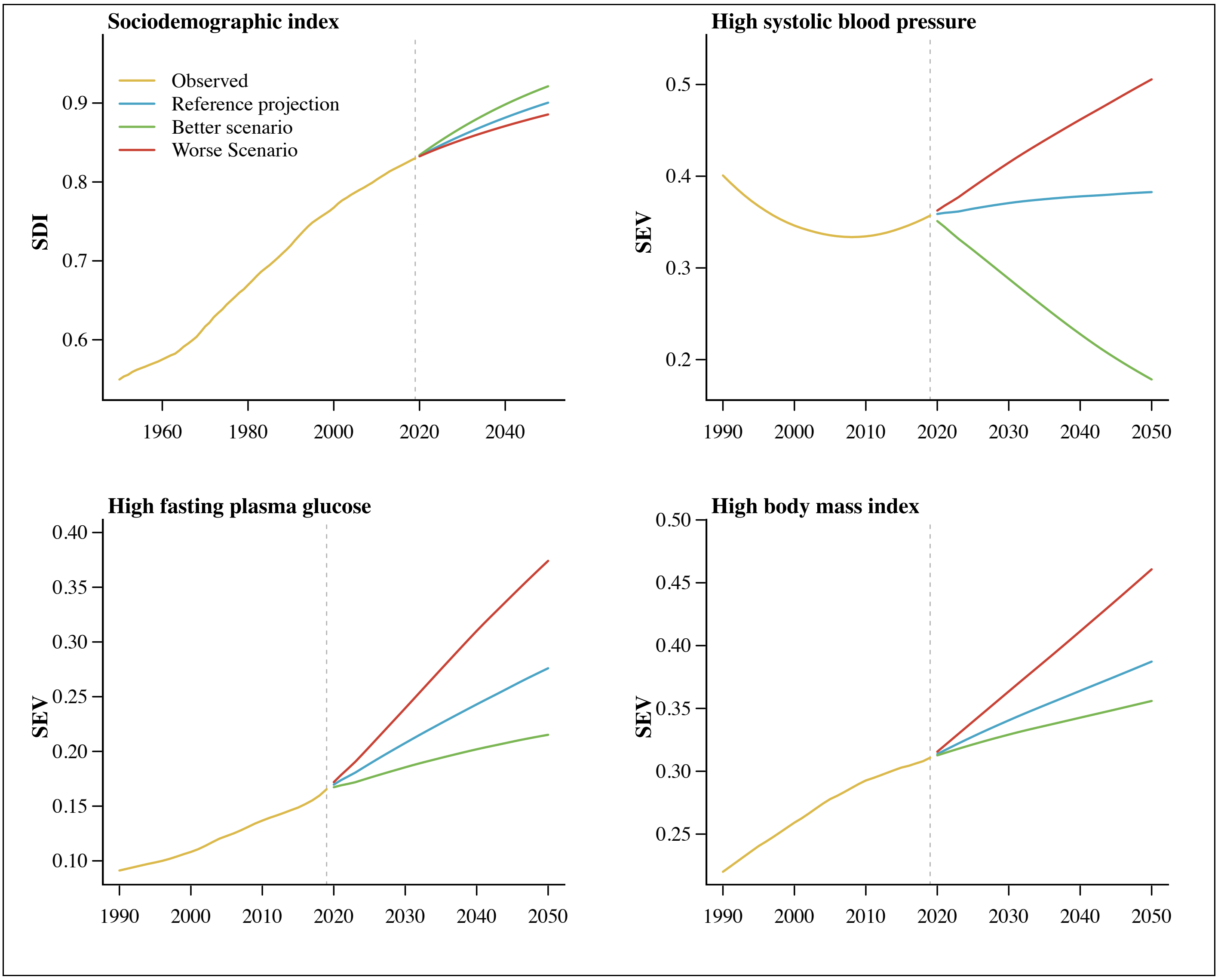

Figure S3: The independent drivers in Europe over time (1990-2050).

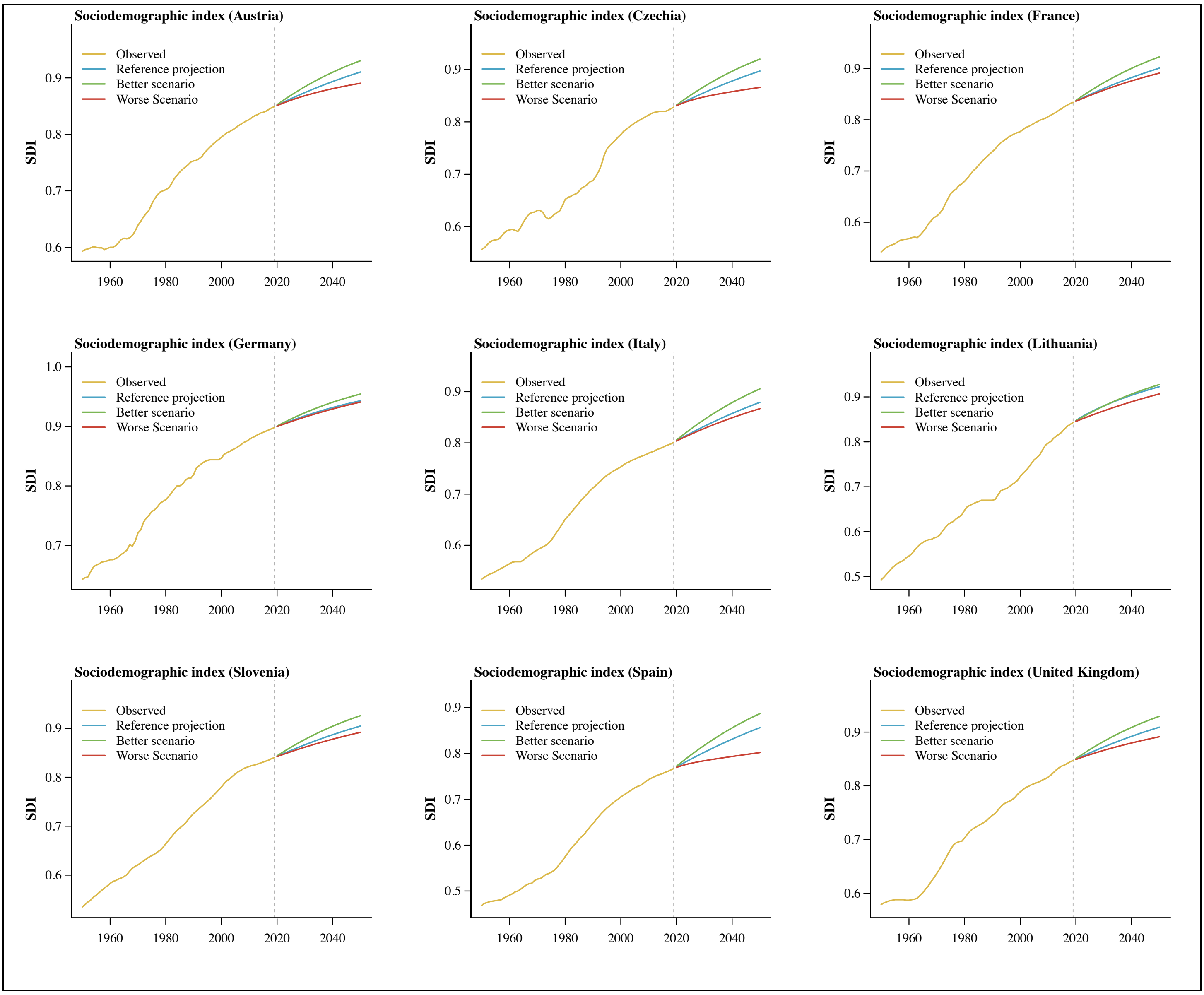

Figure S4: Sociodemographic index in selected European countries over time (1990-2050).

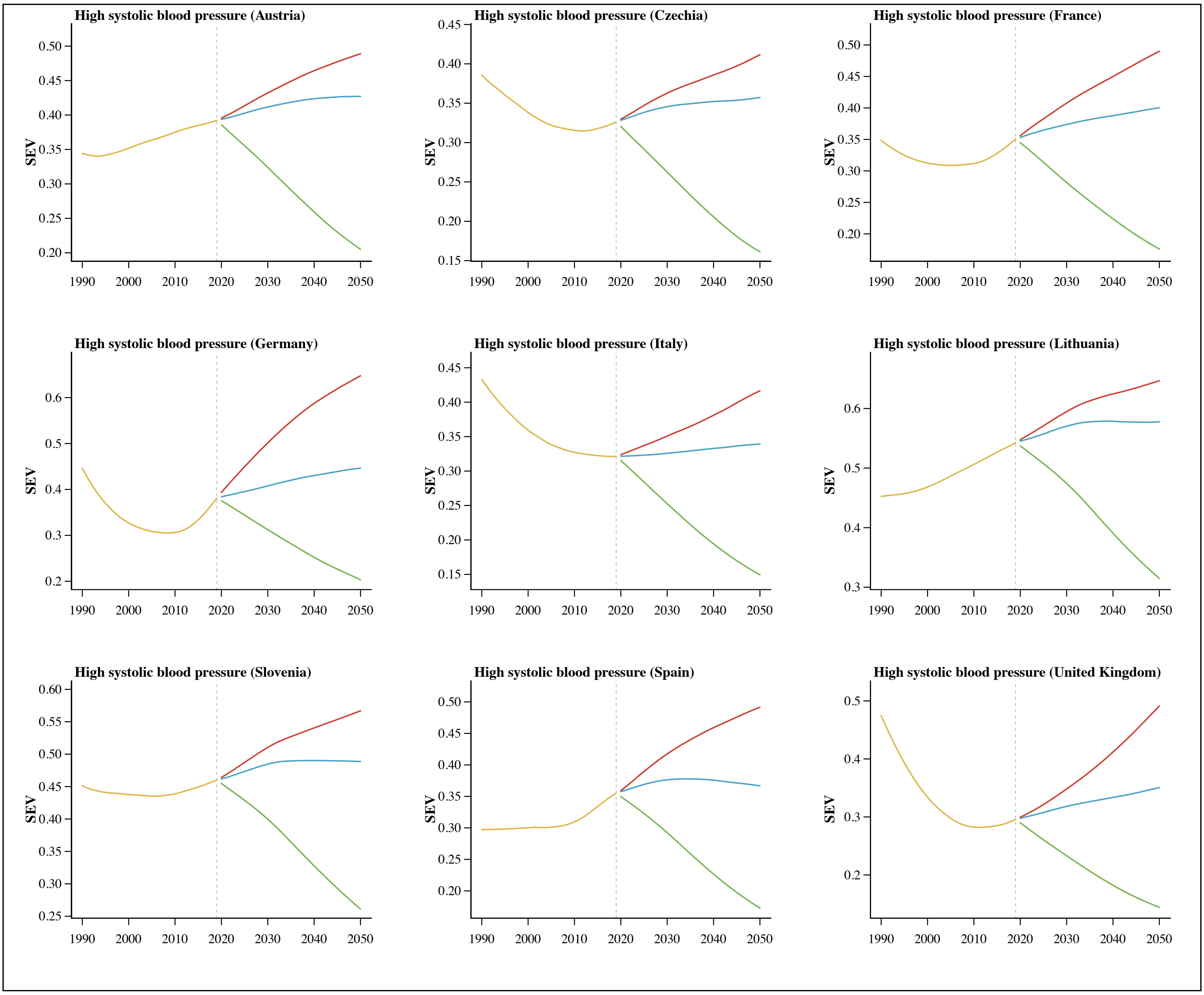

Figure S5: High systolic blood pressure in selected European countries over time (1990-2050).

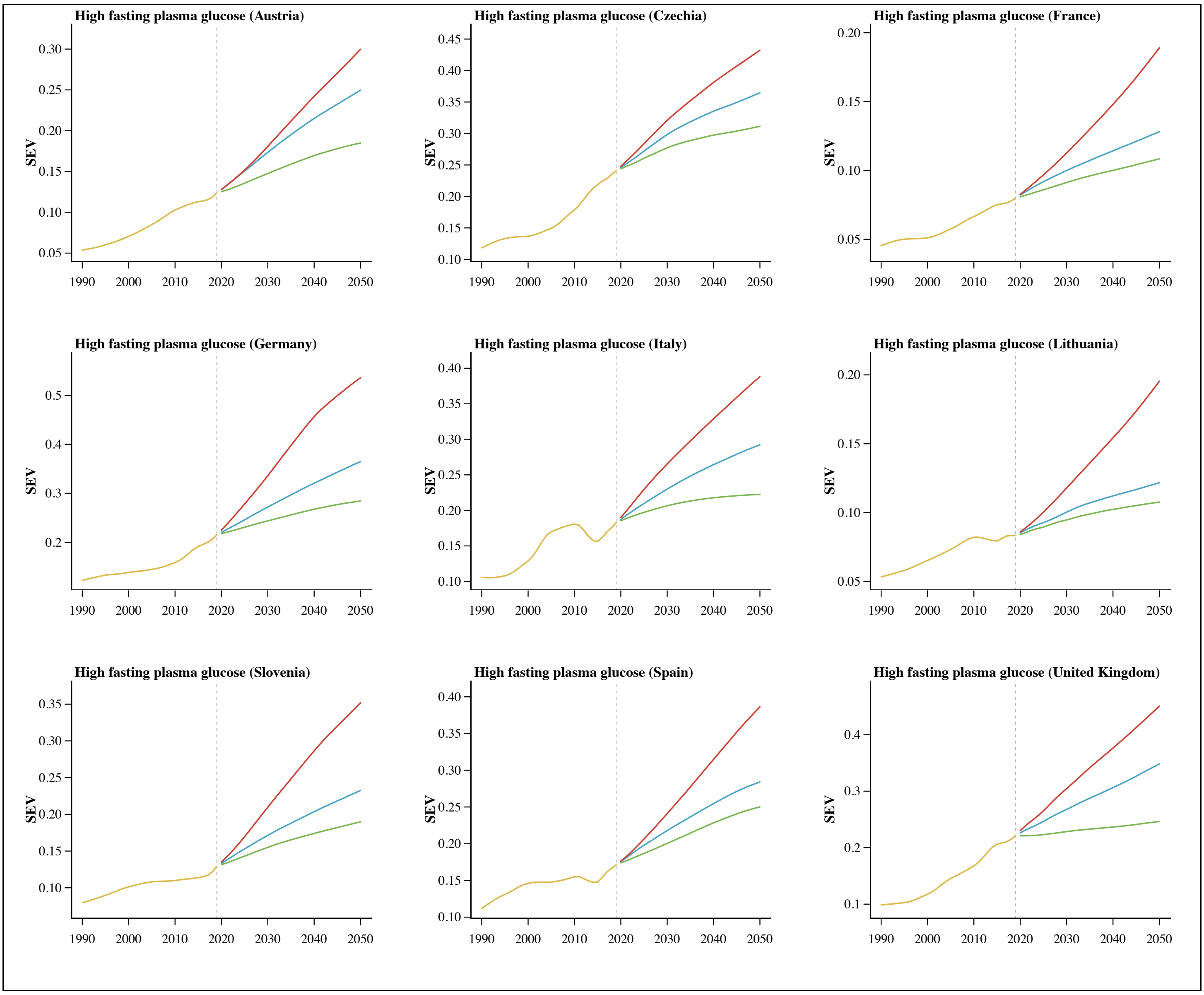

Figure S6: High fasting plasma glucose in selected European countries over time (1990-2050).

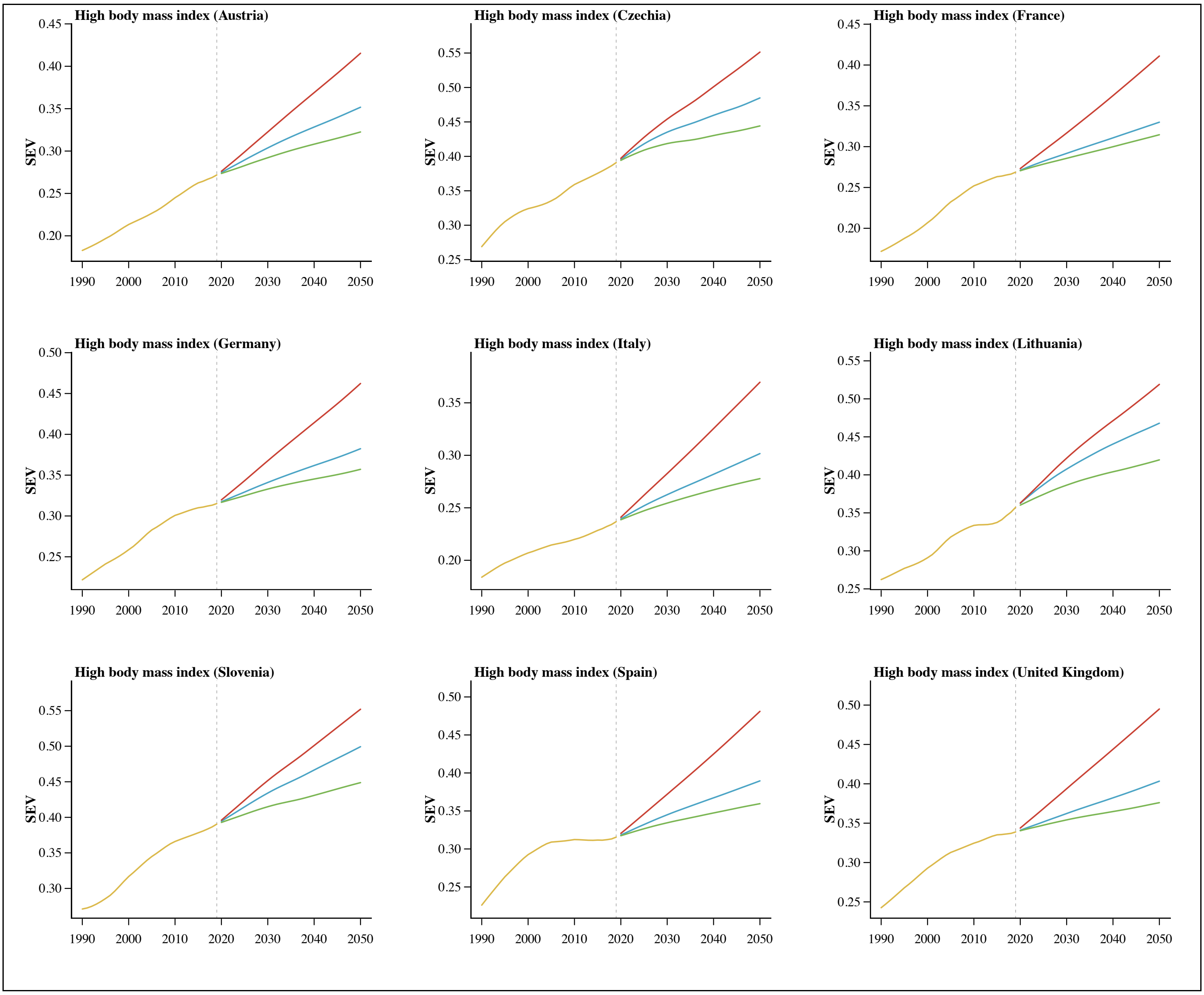

Figure S7: High body mass index in selected European countries over time (1990-2050).

Table S2: Population demography and ICH incidence and mortality by EU country, in 1990, 2019, and 2050 reference forecast.

	1990	2019	2050	Relative Change	
				2019 vs 1990	2050 vs 2019
**EU28**					
Population (m)	475.744928	511.80567	494.749737	1.08	0.97
Females	51.42%	51.08%	50.83%	0.99	1
Age group					
0 to 9	12.61%	10.16%	8.82%	0.81	0.87
10 to 19	13.98%	10.5%	9.18%	0.75	0.87
20 to 39	30.11%	24.65%	21.07%	0.82	0.85
40 to 59	24.17%	28.14%	24.23%	1.16	0.86
60 to 79	15.96%	20.77%	24.95%	1.3	1.2
≥ 80	3.17%	5.79%	11.75%	1.83	2.03
ICH incidence					
Count (k)	175119.3	140374.6	141184.2	0.8	1.01
Adjusted rate*	49.79	26.26	16.82	0.53	0.64
ICH mortality					
Count (k)	169635.2	132428.3	144188.7	0.78	1.09
Adjusted rate*	51.54	24.81	15.04	0.48	0.61
**Austria**					
Population (m)	7.678732	8.879941	8.924192	1.16	1
Females	52.13%	50.81%	50.44%	0.97	0.99
Age group					
0 to 9	11.84%	9.67%	8.38%	0.82	0.87
10 to 19	12.38%	9.7%	8.68%	0.78	0.89
20 to 39	31.61%	26.32%	21.28%	0.83	0.81
40 to 59	24.03%	29.14%	24.45%	1.21	0.84
60 to 79	16.56%	20.01%	24.72%	1.21	1.24
≥ 80	3.58%	5.16%	12.5%	1.44	2.42
ICH incidence					
Count (k)	3112.111	1919.327	1736.952	0.62	0.9

Adjusted rate*	53.43	21.58	10.96	0.4	0.51
ICH mortality					
Count (k)	2141.683	1279.664	1545.767	0.6	1.21
Adjusted rate*	39.22	14.49	8.26	0.37	0.57
**Belgium**					
Population (m)	9.959562	11.510573	12.090658	1.16	1.05
Females	51.12%	50.67%	50.25%	0.99	0.99
Age group					
0 to 9	12.01%	11.11%	9.55%	0.93	0.86
10 to 19	12.72%	11.29%	9.75%	0.89	0.86
20 to 39	30.67%	25.38%	22.23%	0.83	0.88
40 to 59	24.04%	26.98%	24.82%	1.12	0.92
60 to 79	17.06%	19.57%	22.84%	1.15	1.17
≥ 80	3.5%	5.68%	10.81%	1.62	1.9
ICH incidence					
Count (k)	3077.91	2798.153	3424.924	0.91	1.22
Adjusted rate*	40.6	23.93	17.06	0.59	0.71
ICH mortality					
Count (k)	3106.127	2845.975	3473.387	0.92	1.22
Adjusted rate*	43.07	24.46	16.22	0.57	0.66
**Bulgaria**					
Population (m)	8.767776	7.05253	5.187392	0.8	0.74
Females	51.22%	51.49%	50.97%	1.01	0.99
Age group					
0 to 9	13.03%	9.34%	8.47%	0.72	0.91
10 to 19	14.51%	9.24%	8.61%	0.64	0.93
20 to 39	27.28%	23.83%	20.38%	0.87	0.86
40 to 59	25.36%	28.72%	23.17%	1.13	0.81
60 to 79	17.08%	23.55%	30%	1.38	1.27
≥ 80	2.74%	5.31%	9.36%	1.94	1.76
ICH incidence					
Count (k)	11060.649	6261.114	4233.459	0.57	0.68

Adjusted rate*	159.91	84.61	56.76	0.53	0.67
ICH mortality					
Count (k)	12274.404	7607.901	5074.846	0.62	0.67
Adjusted rate*	185.09	103.5	64.66	0.56	0.62
**Croatia**					
Population (m)	4.873707	4.129756	3.333427	0.85	0.81
Females	51.63%	51.39%	50.5%	1	0.98
Age group					
0 to 9	12.71%	9.34%	8.04%	0.73	0.86
10 to 19	13.75%	9.81%	8.62%	0.71	0.88
20 to 39	30.09%	25.07%	20.36%	0.83	0.81
40 to 59	25.71%	27.37%	24.97%	1.06	0.91
60 to 79	15.33%	22.81%	27.33%	1.49	1.2
≥ 80	2.41%	5.6%	10.66%	2.32	1.9
ICH incidence					
Count (k)	2772.953	1536.018	1214.147	0.55	0.79
Adjusted rate*	76.65	35.65	22.3	0.47	0.63
ICH mortality					
Count (k)	2823.029	1607.482	1156.352	0.57	0.72
Adjusted rate*	85.98	37.71	20.3	0.44	0.54
**Cyprus**					
Population (m)	0.788499	1.228831	1.391791	1.56	1.13
Females	49.38%	49.9%	49.73%	1.01	1
Age group					
0 to 9	17.31%	10.93%	8.22%	0.63	0.75
10 to 19	15.5%	10.31%	8.28%	0.67	0.8
20 to 39	32.87%	32.83%	22.25%	1	0.68
40 to 59	20.97%	26.81%	25.3%	1.28	0.94
60 to 79	11.34%	15.98%	27.52%	1.41	1.72
≥ 80	2%	3.15%	8.43%	1.57	2.68
ICH incidence					
Count (k)	217.9958	262.9701	379.0055	1.21	1.44

Adjusted rate*	49.26	28.53	19.34	0.58	0.68
ICH mortality					
Count (k)	237.5018	215.7797	375.8608	0.91	1.74
Adjusted rate*	58.89	25.49	18.19	0.43	0.71
**Czechia**					
Population (m)	10.301193	10.536874	10.577128	1.02	1
Females	51.46%	50.75%	48.63%	0.99	0.96
Age group					
0 to 9	12.99%	10.62%	10.44%	0.82	0.98
10 to 19	16.6%	9.73%	9.66%	0.59	0.99
20 to 39	27.92%	24.73%	24.67%	0.89	1
40 to 59	24.73%	28.76%	23.08%	1.16	0.8
60 to 79	15.32%	22.05%	24.52%	1.44	1.11
≥ 80	2.44%	4.1%	7.63%	1.68	1.86
ICH incidence					
Count (k)	4165.144	2686.877	2218.721	0.65	0.83
Adjusted rate*	60.46	27.39	16.08	0.45	0.59
ICH mortality					
Count (k)	4121.163	1930.899	1497.594	0.47	0.78
Adjusted rate*	65.3	20.38	10.09	0.31	0.5
**Denmark**					
Population (m)	5.144621	5.79588	6.445112	1.13	1.11
Females	50.71%	50.28%	49.99%	0.99	0.99
Age group					
0 to 9	10.89%	10.55%	9.9%	0.97	0.94
10 to 19	13.29%	11.78%	10.64%	0.89	0.9
20 to 39	29.94%	25.06%	23.08%	0.84	0.92
40 to 59	25.49%	26.89%	26.21%	1.05	0.97
60 to 79	16.67%	21.08%	20.66%	1.26	0.98
≥ 80	3.71%	4.64%	9.5%	1.25	2.05
ICH incidence					
Count (k)	1754.509	1220.369	1285.992	0.7	1.05

Adjusted rate*	43.49	21.94	13.46	0.5	0.61
ICH mortality					
Count (k)	1718.219	1177.809	1489.72	0.69	1.26
Adjusted rate*	44.06	21.53	13.92	0.49	0.65
**Estonia**					
Population (m)	1.570674	1.32704	1.171699	0.84	0.88
Females	53.2%	52.71%	51.3%	0.99	0.97
Age group					
0 to 9	15.15%	10.89%	9.48%	0.72	0.87
10 to 19	14.05%	10.22%	9.35%	0.73	0.91
20 to 39	29.15%	25.87%	22%	0.89	0.85
40 to 59	24.44%	26.68%	22.49%	1.09	0.84
60 to 79	14.65%	20.6%	26.58%	1.41	1.29
≥ 80	2.55%	5.74%	10.11%	2.25	1.76
ICH incidence					
Count (k)	784.7706	316.1426	238.9713	0.4	0.76
Adjusted rate*	69.7	24.28	14.36	0.35	0.59
ICH mortality					
Count (k)	608.5734	200.682	197.9519	0.33	0.99
Adjusted rate*	60.48	15.65	10.21	0.26	0.65
**Finland**					
Population (m)	4.986543	5.521536	5.460919	1.11	0.99
Females	51.48%	50.63%	50.31%	0.98	0.99
Age group					
0 to 9	12.79%	10.31%	8.81%	0.81	0.85
10 to 19	12.59%	10.93%	9.17%	0.87	0.84
20 to 39	30.46%	25.02%	21.57%	0.82	0.86
40 to 59	25.69%	25.14%	26.05%	0.98	1.04
60 to 79	15.65%	23.03%	23.4%	1.47	1.02
≥ 80	2.83%	5.57%	11.01%	1.97	1.98
ICH incidence					
Count (k)	1303.074	1643.643	1783.799	1.26	1.09

Adjusted rate*	37.56	28.12	19.1	0.75	0.68
ICH mortality					
Count (k)	1139.294	1152.504	1621.217	1.01	1.41
Adjusted rate*	35.19	19.62	15.88	0.56	0.81
**France**					
Population (m)	56.412895	64.39976	65.827068	1.14	1.02
Females	51.34%	51.64%	52.59%	1.01	1.02
Age group					
0 to 9	13.38%	11.55%	9.92%	0.86	0.86
10 to 19	14.26%	12.23%	10.83%	0.86	0.89
20 to 39	30%	23.48%	20.75%	0.78	0.88
40 to 59	23.13%	25.97%	23.93%	1.12	0.92
60 to 79	15.49%	20.47%	22.81%	1.32	1.11
≥ 80	3.75%	6.3%	11.76%	1.68	1.87
ICH incidence					
Count (k)	13895.52	13953.88	14717.29	1	1.05
Adjusted rate*	32.98	19.92	12.52	0.6	0.63
ICH mortality					
Count (k)	14726.7	12109.99	13771.64	0.82	1.14
Adjusted rate*	37.18	16.78	9.64	0.45	0.57
**Germany**					
Population (m)	79.370198	83.148137	78.93223	1.05	0.95
Females	51.89%	50.66%	50.26%	0.98	0.99
Age group					
0 to 9	11%	9.2%	8.45%	0.84	0.92
10 to 19	10.84%	9.23%	8.76%	0.85	0.95
20 to 39	31.76%	24.57%	21.41%	0.77	0.87
40 to 59	26.09%	28.57%	24.03%	1.1	0.84
60 to 79	16.59%	21.68%	23.95%	1.31	1.1
≥ 80	3.72%	6.75%	13.41%	1.81	1.99
ICH incidence					
Count (k)	23048.39	19015.19	18018.09	0.83	0.95

Adjusted rate*	36.93	20.06	12.02	0.54	0.6
ICH mortality					
Count (k)	22186.76	15532.91	17236.97	0.7	1.11
Adjusted rate*	37.87	16.17	10.61	0.43	0.66
**Greece**					
Population (m)	10.302257	10.574023	9.144953	1.03	0.86
Females	50.56%	50.99%	51.03%	1.01	1
Age group					
0 to 9	12.01%	9.13%	7.95%	0.76	0.87
10 to 19	14.78%	10.38%	8.35%	0.7	0.8
20 to 39	28.41%	23.12%	19.2%	0.81	0.83
40 to 59	24.92%	29.12%	24.1%	1.17	0.83
60 to 79	16.68%	21.52%	27.28%	1.29	1.27
≥ 80	3.19%	6.74%	13.11%	2.11	1.95
ICH incidence					
Count (k)	6205.673	5731.924	6318.864	0.92	1.1
Adjusted rate*	80.19	47.15	32.71	0.59	0.69
ICH mortality					
Count (k)	7136.721	6475.747	7474.821	0.91	1.15
Adjusted rate*	97.76	52.8	35.81	0.54	0.68
**Hungary**					
Population (m)	10.37599	9.771796	8.817396	0.94	0.9
Females	51.98%	52.12%	51.17%	1	0.98
Age group					
0 to 9	12.19%	9.48%	8.85%	0.78	0.93
10 to 19	15.64%	10.06%	9.5%	0.64	0.94
20 to 39	28.05%	25.22%	21.98%	0.9	0.87
40 to 59	25.12%	28.74%	25.44%	1.14	0.89
60 to 79	16.43%	22.04%	26.18%	1.34	1.19
≥ 80	2.57%	4.47%	8.05%	1.74	1.8
ICH incidence					
Count (k)	6932.755	3346.614	2425.721	0.48	0.72

Adjusted rate*	92.68	36.19	21.04	0.39	0.58
ICH mortality					
Count (k)	6777.829	2527.976	1764.085	0.37	0.7
Adjusted rate*	99.46	28	13.61	0.28	0.49
**Ireland**					
Population (m)	3.485375	4.896023	5.724686	1.4	1.17
Females	50.31%	50.47%	50.26%	1	1
Age group					
0 to 9	17.24%	13.48%	10.51%	0.78	0.78
10 to 19	19.32%	13.48%	10.45%	0.7	0.78
20 to 39	28.2%	26.58%	22.99%	0.94	0.86
40 to 59	19.92%	27.07%	24.04%	1.36	0.89
60 to 79	13.07%	16.05%	23.6%	1.23	1.47
≥ 80	2.24%	3.34%	8.41%	1.49	2.52
ICH incidence					
Count (k)	774.0937	737.3577	1074.7942	0.95	1.46
Adjusted rate*	36.43	19.6	13.87	0.54	0.71
ICH mortality					
Count (k)	671.8929	492	858.1725	0.73	1.74
Adjusted rate*	35.07	14.42	9.92	0.41	0.69
**Italy**					
Population (m)	56.756562	59.727934	52.250485	1.05	0.87
Females	51.49%	51.3%	50.81%	1	0.99
Age group					
0 to 9	10.22%	8.31%	7.27%	0.81	0.87
10 to 19	13.97%	9.56%	7.77%	0.68	0.81
20 to 39	29.66%	21.81%	18.12%	0.74	0.83
40 to 59	25.43%	30.8%	23.54%	1.21	0.76
60 to 79	17.41%	22.22%	27.54%	1.28	1.24
≥ 80	3.3%	7.29%	15.77%	2.21	2.16
ICH incidence					
Count (k)	17078.72	16553.91	17813.75	0.97	1.08

Adjusted rate*	38.77	23.41	16.35	0.6	0.7
ICH mortality					
Count (k)	16963.56	18305.61	23811.8	1.08	1.3
Adjusted rate*	40.62	24.68	16.5	0.61	0.67
**Latvia**					
Population (m)	2.689385	1.91656	1.433731	0.71	0.75
Females	53.86%	53.85%	52.12%	1	0.97
Age group					
0 to 9	14.79%	10.35%	9.46%	0.7	0.91
10 to 19	13.31%	10%	9.06%	0.75	0.91
20 to 39	29.25%	24.35%	22.71%	0.83	0.93
40 to 59	24.86%	27.4%	22.53%	1.1	0.82
60 to 79	14.95%	21.83%	26.41%	1.46	1.21
≥ 80	2.83%	6.08%	9.84%	2.15	1.62
ICH incidence					
Count (k)	1774.3137	800.9878	495.001	0.45	0.62
Adjusted rate*	93.95	40.01	24.64	0.43	0.62
ICH mortality					
Count (k)	1558.6344	671.6937	421.1001	0.43	0.63
Adjusted rate*	90.64	33.59	17.75	0.37	0.53
**Lithuania**					
Population (m)	3.785847	2.849084	2.187556	0.75	0.77
Females	52.97%	53.25%	51.84%	1.01	0.97
Age group					
0 to 9	15.4%	10.33%	9.46%	0.67	0.92
10 to 19	14.46%	9.38%	8.96%	0.65	0.96
20 to 39	30.49%	24.93%	22.58%	0.82	0.91
40 to 59	23.51%	28.33%	22.74%	1.21	0.8
60 to 79	13.34%	20.96%	25.09%	1.57	1.2
≥ 80	2.81%	6.07%	11.16%	2.16	1.84
ICH incidence					
Count (k)	1214.6491	979.2487	783.1323	0.81	0.8

Adjusted rate*	44.89	32.78	25.3	0.73	0.77
ICH mortality					
Count (k)	753.473	662.8906	530.9564	0.88	0.8
Adjusted rate*	30.94	22.22	15.1	0.72	0.68
**Luxembourg**					
Population (m)	0.381266	0.61997	0.781912	1.63	1.26
Females	51%	49.7%	49.96%	0.97	1.01
Age group					
0 to 9	11.86%	10.72%	8.87%	0.9	0.83
10 to 19	11.36%	10.67%	8.99%	0.94	0.84
20 to 39	32.57%	29.42%	22.88%	0.9	0.78
40 to 59	25.34%	29.27%	26.24%	1.16	0.9
60 to 79	15.93%	15.96%	23.77%	1	1.49
≥ 80	2.94%	3.96%	9.24%	1.35	2.33
ICH incidence					
Count (k)	135.765	105.4741	152.8779	0.78	1.45
Adjusted rate*	50.93	21.36	12.9	0.42	0.6
ICH mortality					
Count (k)	153.4159	103.2612	176.3077	0.67	1.71
Adjusted rate*	61.38	22.26	13.83	0.36	0.62
**Malta**					
Population (m)	0.365397	0.503636	0.522734	1.38	1.04
Females	50.54%	48.45%	47.39%	0.96	0.98
Age group					
0 to 9	15.27%	9.1%	6.66%	0.6	0.73
10 to 19	15.53%	8.56%	7.25%	0.55	0.85
20 to 39	29.84%	32.38%	19.71%	1.09	0.61
40 to 59	24.53%	25.12%	26.47%	1.02	1.05
60 to 79	13.13%	20.37%	29.56%	1.55	1.45
≥ 80	1.7%	4.47%	10.35%	2.63	2.32
ICH incidence					
Count (k)	103.2656	113.915	130.7871	1.1	1.15

Adjusted rate*	59.58	24.85	14.91	0.42	0.6
ICH mortality					
Count (k)	107.6291	86.36605	91.88118	0.8	1.06
Adjusted rate*	74.81	19.63	9.62	0.26	0.49
**Netherlands**					
Population (m)	14.944548	17.363261	17.897026	1.16	1.03
Females	50.58%	50.33%	49.96%	1	0.99
Age group					
0 to 9	12.21%	10.25%	9.12%	0.84	0.89
10 to 19	13.23%	11.56%	10.08%	0.87	0.87
20 to 39	33%	25.09%	21.88%	0.76	0.87
40 to 59	24.2%	27.4%	25.57%	1.13	0.93
60 to 79	14.47%	21.02%	22.3%	1.45	1.06
≥ 80	2.89%	4.67%	11.05%	1.62	2.37
ICH incidence					
Count (k)	3730.021	3720.432	4622.558	1	1.24
Adjusted rate*	37.12	22.31	15.74	0.6	0.71
ICH mortality					
Count (k)	3058.692	3145.122	4558.671	1.03	1.45
Adjusted rate*	33.57	19.41	13.89	0.58	0.72
**Poland**					
Population (m)	38.064257	38.4936	34.932341	1.01	0.91
Females	51.27%	51.61%	51.38%	1.01	1
Age group					
0 to 9	16.45%	10.14%	8.27%	0.62	0.82
10 to 19	16.01%	9.88%	8.54%	0.62	0.86
20 to 39	30.57%	28.09%	21.12%	0.92	0.75
40 to 59	22.05%	26.84%	24.16%	1.22	0.9
60 to 79	12.91%	20.7%	28.79%	1.6	1.39
≥ 80	2.01%	4.35%	9.12%	2.16	2.1
ICH incidence					
Count (k)	14605.85	11144.45	10879.65	0.76	0.98

Adjusted rate*	63.76	31.45	21.02	0.49	0.67
ICH mortality					
Count (k)	13840.45	9891.14	10788.98	0.71	1.09
Adjusted rate*	68.75	29.51	19.12	0.43	0.65
**Portugal**					
Population (m)	10.007344	10.289926	9.261307	1.03	0.9
Females	51.83%	52.79%	52.15%	1.02	0.99
Age group					
0 to 9	12.47%	8.69%	7.77%	0.7	0.89
10 to 19	16.38%	10.27%	8.48%	0.63	0.83
20 to 39	28.83%	22.77%	19.27%	0.79	0.85
40 to 59	23.33%	29.71%	24.2%	1.27	0.81
60 to 79	16.22%	22.03%	27.16%	1.36	1.23
≥ 80	2.78%	6.53%	13.11%	2.35	2.01
ICH incidence					
Count (k)	6390.674	3622.889	3563.938	0.57	0.98
Adjusted rate*	90.04	31.82	20.97	0.35	0.66
ICH mortality					
Count (k)	7458.235	4235.916	4109.237	0.57	0.97
Adjusted rate*	112.22	37.37	21.71	0.33	0.58
**Romania**					
Population (m)	22.836232	19.52421	17.457212	0.85	0.89
Females	51.18%	51.59%	51.35%	1.01	1
Age group					
0 to 9	14.9%	10.57%	9.54%	0.71	0.9
10 to 19	16.78%	10.71%	9.87%	0.64	0.92
20 to 39	28.89%	25.04%	22.29%	0.87	0.89
40 to 59	23.57%	28.61%	23.42%	1.21	0.82
60 to 79	13.96%	20.51%	26.49%	1.47	1.29
≥ 80	1.89%	4.57%	8.39%	2.42	1.84
ICH incidence					
Count (k)	18394.371	12286.225	9749.624	0.67	0.79

Adjusted rate*	123.56	68.11	40.42	0.55	0.59
ICH mortality					
Count (k)	15092.419	12916.282	9077.858	0.86	0.7
Adjusted rate*	112.63	73.59	36.33	0.65	0.49
**Slovakia**					
Population (m)	5.261306	5.453929	5.186965	1.04	0.95
Females	51.16%	51.17%	50.96%	1	1
Age group					
0 to 9	16.22%	10.72%	8.82%	0.66	0.82
10 to 19	17.24%	9.91%	8.94%	0.57	0.9
20 to 39	30.61%	27.99%	21.92%	0.91	0.78
40 to 59	21.23%	28.43%	23.84%	1.34	0.84
60 to 79	12.8%	19.62%	28.1%	1.53	1.43
≥ 80	1.89%	3.34%	8.38%	1.77	2.51
ICH incidence					
Count (k)	2112.922	1514.225	1514.241	0.72	1
Adjusted rate*	70.99	32.95	21.25	0.46	0.64
ICH mortality					
Count (k)	2251.466	1307.826	1244.712	0.58	0.95
Adjusted rate*	83.46	30.13	16.02	0.36	0.53
**Slovenia**					
Population (m)	1.986025	2.112904	2.003256	1.06	0.95
Females	51.77%	49.92%	49.58%	0.96	0.99
Age group					
0 to 9	13.25%	10.25%	9.04%	0.77	0.88
10 to 19	14.91%	9.47%	8.9%	0.64	0.94
20 to 39	31.73%	24.72%	20.77%	0.78	0.84
40 to 59	24.29%	29%	22.49%	1.19	0.78
60 to 79	13.48%	21.31%	26.93%	1.58	1.26
≥ 80	2.34%	5.24%	11.88%	2.24	2.27
ICH incidence					
Count (k)	649.0191	511.6754	579.8748	0.79	1.13

Adjusted rate*	52.03	24.27	15.82	0.47	0.65
ICH mortality					
Count (k)	561.2572	460.0494	492.4632	0.82	1.07
Adjusted rate*	50.06	22.56	12.56	0.45	0.56
**Spain**					
Population (m)	38.88989	47.131376	44.219563	1.21	0.94
Females	51.03%	50.98%	51.17%	1	1
Age group					
0 to 9	11.77%	9.3%	7.44%	0.79	0.8
10 to 19	16.67%	10.37%	7.83%	0.62	0.76
20 to 39	29.87%	23.39%	18.52%	0.78	0.79
40 to 59	22.68%	31.38%	23.49%	1.38	0.75
60 to 79	16.05%	19.49%	28.64%	1.21	1.47
≥ 80	2.96%	6.07%	14.08%	2.05	2.32
ICH incidence					
Count (k)	12494.73	11186.14	10758.65	0.9	0.96
Adjusted rate*	45.77	22.09	11.93	0.48	0.54
ICH mortality					
Count (k)	13006.27	11171.24	11868.86	0.86	1.06
Adjusted rate*	51.61	21.83	10.97	0.42	0.5
**Sweden**					
Population (m)	8.548406	10.267923	11.902036	1.2	1.16
Females	50.6%	49.71%	49.45%	0.98	0.99
Age group					
0 to 9	12.11%	11.89%	10.16%	0.98	0.85
10 to 19	12.4%	11.33%	9.92%	0.91	0.88
20 to 39	27.73%	26.1%	23.5%	0.94	0.9
40 to 59	24.96%	25.2%	25.24%	1.01	1
60 to 79	18.54%	20.33%	21.82%	1.1	1.07
≥ 80	4.26%	5.16%	9.37%	1.21	1.82
ICH incidence					
Count (k)	2847.836	2802.439	3378.615	0.98	1.21

Adjusted rate*	38.85	27.51	20.04	0.71	0.73
ICH mortality					
Count (k)	2154.08	1919.721	2118.137	0.89	1.1
Adjusted rate*	30.33	18.81	11.08	0.62	0.59
**United Kingdom**					
Population (m)	57.210441	66.778657	71.684962	1.17	1.07
Females	51.41%	50.62%	50.12%	0.98	0.99
Age group					
0 to 9	13.04%	11.96%	9.77%	0.92	0.82
10 to 19	12.78%	11.41%	9.75%	0.89	0.85
20 to 39	29.76%	26.28%	22.91%	0.88	0.87
40 to 59	23.63%	26.18%	25.18%	1.11	0.96
60 to 79	17.18%	19.13%	22.2%	1.11	1.16
≥ 80	3.61%	5.04%	10.19%	1.4	2.02
ICH incidence					
Count (k)	14481.57	13603.01	17690.74	0.94	1.3
Adjusted rate*	32.16	20.96	15.89	0.65	0.76
ICH mortality					
Count (k)	13005.77	12393.85	17359.32	0.95	1.4
Adjusted rate*	30.24	19.34	14.04	0.64	0.73

Table S3: Number of ICH cases in 2019 and 2050 and percentage change in counts and age-standardised rates by country.

	Number of new ICH cases				Percentage change (2050 vs 2019)	
	2019	2050 forecast	2050 better scenario	2050 worse scenario	Count	Age-standardised rates
EU28	140,388 (130,253 to 151,866)	141,202 (120,648 to 166,489)	126,606 (108,909 to 148,377)	163,861 (141,599 to 190,340)	0.6 (-7.4 to 9.6)	-35.9 (-32 to -38.8)
Austria	1,919 (1,787 to 2,089)	1,737 (1,478 to 2,057)	1,325 (1,149 to 1,553)	1,786 (1,545 to 2,087)	-9.5 (-17.3 to -1.5)	-49.2 (-45.2 to -51.7)
Belgium	2,798 (2,574 to 3,064)	3,425 (2,902 to 4,115)	3,102 (2,600 to 3,764)	3,856 (3,284 to 4,632)	22.4 (12.8 to 34.3)	-28.7 (-23.2 to -32.7)
Bulgaria	6,261 (5,838 to 6,720)	4,233 (3,738 to 4,807)	3,874 (3,413 to 4,433)	4,821 (4,244 to 5,478)	-32.4 (-36 to -28.5)	-32.9 (-28.7 to -36.1)
Croatia	1,536 (1,426 to 1,642)	1,214 (1,053 to 1,420)	1,001 (870 to 1,164)	1,329 (1,171 to 1,521)	-21 (-26.1 to -13.5)	-37.4 (-33 to -40.5)
Cyprus	263 (244 to 287)	379 (329 to 441)	360 (310 to 422)	425 (368 to 492)	44.1 (34.9 to 53.4)	-32.2 (-27.4 to -35.5)
Czechia	2,687 (2,507 to 2,888)	2,219 (1,909 to 2,588)	1,948 (1,693 to 2,257)	2,540 (2,216 to 2,915)	-17.4 (-23.8 to -10.4)	-41.3 (-37.3 to -44.2)
Denmark	1,221 (1,132 to 1,329)	1,286 (1,084 to 1,531)	1,162 (988 to 1,390)	1,419 (1,210 to 1,691)	5.3 (-4.2 to 15.2)	-38.7 (-33.7 to -42.5)
Estonia	316 (296 to 336)	239 (212 to 274)	237 (212 to 267)	309 (279 to 343)	-24.4 (-28.6 to -18.6)	-40.9 (-37.5 to -43.1)
Finland	1,644 (1,527 to 1,786)	1,784 (1,496 to 2,181)	1,696 (1,450 to 2,027)	2,047 (1,764 to 2,403)	8.5 (-2 to 22.1)	-32.1 (-26.9 to -36.1)
France	13,955 (12,856 to 15,269)	14,719 (12,301 to 18,048)	12,968 (10,977 to 15,632)	16,654 (14,195 to 19,935)	5.5 (-4.3 to 18.2)	-37.1 (-32.8 to -40.5)
Germany	19,016 (17,626 to 20,697)	18,020 (15,256 to 21,434)	16,994 (14,397 to 20,188)	21,604 (18,650 to 25,227)	-5.2 (-13.4 to 3.6)	-40.1 (-35.7 to -43.4)
Greece	5,732 (5,266 to 6,328)	6,319 (5,220 to 7,776)	5,920 (4,886 to 7,149)	7,259 (6,041 to 8,842)	10.2 (-0.9 to 22.9)	-30.6 (-24.8 to -34.8)
Hungary	3,347 (3,124 to 3,581)	2,426 (2,159 to 2,736)	1,943 (1,726 to 2,207)	3,165 (2,826 to 3,574)	-27.5 (-30.9 to -23.6)	-41.9 (-38.9 to -43.9)
Ireland	737	1,075	1,009	1,319	45.7	-29.2

	(687 to 796)	(936 to 1,252)	(880 to 1,187)	(1,164 to 1,511)	(36.3 to 57.3)	(-24.8 to -32.6)
Italy	16,557	17,812	13,862	18,105	7.6	-30.2
	(15,245 to 17,979)	(15,325 to 20,723)	(11,791 to 16,056)	(15,647 to 20,809)	(0.5 to 15.3)	(-25.5 to -34)
Latvia	801	495	482	599	-38.2	-38.4
	(747 to 855)	(437 to 571)	(422 to 558)	(530 to 683)	(-41.5 to -33.2)	(-34.4 to -40.4)
Lithuania	979	783	703	835	-20	-22.8
	(919 to 1,041)	(698 to 884)	(623 to 800)	(746 to 940)	(-24.1 to -15.1)	(-19.4 to -25.9)
Luxembourg	105	153	136	180	45	-39.6
	(97 to 116)	(129 to 186)	(114 to 164)	(153 to 216)	(32.8 to 60.7)	(-34 to -43.3)
Malta	114	131	109	150	14.8	-40
	(106 to 125)	(114 to 150)	(94 to 128)	(131 to 175)	(7.6 to 20.5)	(-36.2 to -43.3)
Netherlands	3,721	4,623	4,517	5,548	24.2	-29.4
	(3,448 to 4,042)	(3,910 to 5,512)	(3,829 to 5,385)	(4,730 to 6,666)	(13.4 to 36.4)	(-23.7 to -33.5)
Poland	11,144	10,878	9,051	11,472	-2.4	-33.2
	(10,385 to 11,927)	(9,566 to 12,548)	(7,903 to 10,594)	(10,126 to 13,315)	(-7.9 to 5.2)	(-29.4 to -36.2)
Portugal	3,623	3,564	2,923	3,744	-1.6	-34.1
	(3,359 to 3,939)	(3,086 to 4,147)	(2,494 to 3,417)	(3,220 to 4,317)	(-8.1 to 5.3)	(-30.1 to -37.7)
Romania	12,287	9,750	9,657	12,246	-20.6	-40.7
	(11,389 to 13,184)	(8,461 to 11,365)	(8,478 to 11,012)	(10,861 to 13,916)	(-25.7 to -13.8)	(-36.5 to -43.5)
Slovakia	1,514	1,514	1,235	1,628	0	-35.5
	(1,412 to 1,617)	(1,344 to 1,708)	(1,093 to 1,408)	(1,449 to 1,847)	(-4.8 to 5.6)	(-31.9 to -38.3)
Slovenia	512	580	495	672	13.3	-34.8
	(473 to 553)	(493 to 693)	(419 to 591)	(578 to 799)	(4.1 to 25.3)	(-29.9 to -38.4)
Spain	11,184	10,759	11,196	16,649	-3.8	-46
	(10,313 to 12,290)	(9,106 to 12,703)	(9,436 to 13,281)	(14,292 to 19,522)	(-11.7 to 3.4)	(-41.9 to -49)
Sweden	2,802	3,379	3,210	3,848	20.6	-27.2
	(2,599 to 3,045)	(2,925 to 3,929)	(2,765 to 3,762)	(3,348 to 4,487)	(12.5 to 29)	(-22 to -31)
United Kingdom	13,603	17,692	15,470	19,649	30.1	-24.2
	(12,572 to 14,846)	(15,054 to 20,957)	(13,086 to 18,591)	(16,904 to 23,275)	(19.7 to 41.2)	(-18.9 to -27.6)

Data between brackets are the 95% Uncertainty Inverval.

Table S4: Number of deaths from ICH in 2019 and 2050 and percentage change in counts and age-standardised rates by country.

	Number of ICH deaths				Percentage change (2050 vs 2019)	
	2019	2050 forecast	2050 better scenario	2050 worse scenario	Count	Age-standardised rates
EU28	132,404<br>(126,375 to 139,321)	144,170<br>(122,864 to 172,248)	132,939<br>(112,865 to 158,139)	173,756<br>(148,738 to 206,232)	8.9<br>(-2.8 to 23.6)	-39.4<br>(-31.7 to -45.3)
Austria	1,280<br>(1,218 to 1,357)	1,546<br>(1,304 to 1,864)	1,157<br>(976 to 1,384)	1,711<br>(1,453 to 2,043)	20.8<br>(7 to 37.3)	-43<br>(-35.4 to -48.8)
Belgium	2,845<br>(2,706 to 3,019)	3,474<br>(2,947 to 4,131)	3,521<br>(2,936 to 4,269)	4,379<br>(3,680 to 5,256)	22.1<br>(8.9 to 36.8)	-33.7<br>(-25.4 to -40)
Bulgaria	7,608<br>(7,282 to 7,912)	5,075<br>(4,473 to 5,783)	5,106<br>(4,486 to 5,853)	6,423<br>(5,671 to 7,282)	-33.3<br>(-38.6 to -26.9)	-37.5<br>(-31 to -42.9)
Croatia	1,607<br>(1,536 to 1,678)	1,156<br>(995 to 1,355)	1,035<br>(890 to 1,224)	1,478<br>(1,292 to 1,704)	-28.1<br>(-35.2 to -19.2)	-46.2<br>(-38.8 to -51.2)
Cyprus	216<br>(205 to 229)	376<br>(322 to 443)	357<br>(304 to 426)	420<br>(360 to 497)	74.2<br>(57 to 93.3)	-28.6<br>(-19.3 to -36)
Czechia	1,931<br>(1,847 to 2,028)	1,498<br>(1,293 to 1,753)	1,480<br>(1,270 to 1,718)	2,078<br>(1,782 to 2,426)	-22.5<br>(-30 to -13.5)	-50.5<br>(-44.1 to -55.4)
Denmark	1,178<br>(1,123 to 1,241)	1,489<br>(1,244 to 1,818)	1,203<br>(1,014 to 1,445)	1,491<br>(1,258 to 1,805)	26.5<br>(10.8 to 46.5)	-35.3<br>(-26.1 to -42.2)
Estonia	201<br>(192 to 209)	198<br>(171 to 232)	136<br>(117 to 160)	181<br>(158 to 211)	-1.4<br>(-10.9 to 11.1)	-34.8<br>(-26.4 to -40.4)
Finland	1,152<br>(1,099 to 1,222)	1,621<br>(1,362 to 1,969)	1,290<br>(1,086 to 1,564)	1,562<br>(1,319 to 1,866)	40.7<br>(23.9 to 61.2)	-19.1<br>(-8.5 to -27.3)
France	12,107<br>(11,496 to 12,860)	13,771<br>(11,320 to 17,361)	10,690<br>(8,827 to 13,302)	14,607<br>(12,216 to 17,856)	13.7<br>(-1.5 to 35)	-42.6<br>(-34.6 to -48.5)
Germany	15,532<br>(14,819 to 16,440)	17,239<br>(14,637 to 20,386)	15,210<br>(12,968 to 18,185)	18,383<br>(15,620 to 21,712)	11<br>(-1.2 to 24)	-34.4<br>(-26.6 to -40.8)
Greece	6,476<br>(6,154 to 6,840)	7,475<br>(6,299 to 9,049)	6,695<br>(5,512 to 8,386)	9,061<br>(7,575 to 11,142)	15.4<br>(2.4 to 32.3)	-32.2<br>(-23.4 to -38.7)
Hungary	2,528<br>(2,420 to 2,637)	1,764<br>(1,520 to 2,123)	1,243<br>(1,091 to 1,445)	2,293<br>(2,019 to 2,638)	-30.2<br>(-37.2 to -19.5)	-51.4<br>(-45.3 to -56)
Ireland	492	858	713	923	74.4	-31.2

	(469 to 525)	(727 to 1,023)	(605 to 851)	(793 to 1,095)	(54.9 to 95.1)	(-22.5 to -38.7)
Italy	18,307	23,811	24,192	29,203	30.1	-33.1
	(17,399 to 19,457)	(19,775 to 29,147)	(20,095 to 29,672)	(24,416 to 35,077)	(13.7 to 49.8)	(-21.9 to -41)
Latvia	672	421	317	542	-37.3	-47.2
	(642 to 699)	(362 to 498)	(277 to 367)	(477 to 622)	(-43.7 to -28.7)	(-40.7 to -51.6)
Lithuania	663	531	508	595	-19.9	-32
	(638 to 694)	(466 to 614)	(444 to 589)	(525 to 683)	(-27 to -11.6)	(-24.9 to -37.9)
Luxembourg	103	176	147	197	70.7	-37.9
	(98 to 110)	(147 to 214)	(123 to 182)	(166 to 243)	(50 to 94.6)	(-28.9 to -45)
Malta	86	92	74	96	6.4	-51
	(83 to 92)	(79 to 108)	(63 to 87)	(83 to 111)	(-3.8 to 17.5)	(-45.3 to -55.5)
Netherlands	3,144	4,558	4,020	5,052	45	-28.4
	(2,993 to 3,330)	(3,842 to 5,474)	(3,357 to 4,831)	(4,238 to 6,052)	(28.3 to 64.4)	(-18.7 to -35.5)
Poland	9,893	10,791	9,013	11,800	9.1	-35.2
	(9,486 to 10,338)	(9,375 to 12,579)	(7,865 to 10,495)	(10,369 to 13,595)	(-1.2 to 21.7)	(-28 to -40.9)
Portugal	4,236	4,109	2,968	3,993	-3	-41.9
	(4,043 to 4,448)	(3,509 to 4,858)	(2,513 to 3,525)	(3,429 to 4,691)	(-13.2 to 9.2)	(-34.3 to -47.9)
Romania	12,917	9,078	9,919	12,637	-29.7	-50.6
	(12,364 to 13,482)	(7,965 to 10,411)	(8,676 to 11,416)	(11,122 to 14,406)	(-35.6 to -22.8)	(-45.5 to -54.7)
Slovakia	1,308	1,245	1,134	1,610	-4.8	-46.8
	(1,254 to 1,368)	(1,090 to 1,427)	(999 to 1,290)	(1,420 to 1,819)	(-13.1 to 4.4)	(-41.5 to -51.4)
Slovenia	460	492	501	656	7	-44.3
	(439 to 485)	(422 to 583)	(428 to 601)	(565 to 772)	(-4 to 20.3)	(-36.3 to -50.3)
Spain	11,170	11,869	10,520	18,630	6.3	-49.7
	(10,613 to 11,864)	(9,998 to 14,241)	(8,849 to 12,614)	(15,920 to 22,153)	(-5.8 to 20)	(-42.5 to -54.8)
Sweden	1,920	2,119	2,584	2,947	10.3	-41.1
	(1,828 to 2,047)	(1,787 to 2,553)	(2,152 to 3,138)	(2,471 to 3,535)	(-2.2 to 24.7)	(-33 to -47.3)
United Kingdom	12,394	17,362	17,190	20,817	40.1	-27.4
	(11,821 to 13,159)	(14,736 to 20,867)	(14,416 to 21,046)	(17,642 to 25,089)	(24.7 to 58.6)	(-18.3 to -34.5)

Data between brackets are the 95% Uncertainty Inverval.

Framework performance – out-of-sample predictive validity

We appraised our forecasting framework by fitting models to data from the period of 1990 to 2009 and then projecting ICH outcomes for the period from 2010 to 2019. Predictions for the period of 2010 to 2019 were contrasted with actual observed data from the same years. We employed the mean coefficient of variation as measured by the root-mean-squared error (RMSE) to assess performance.

Our model's performance was benchmarked against the Lee-Carter approach and the average annulaised rate of change (ARC) exprapolation method by age-country-year. The figures below showcase the results from these analyses by different age and sex groups and EU country. Overall, our forecasting framework outperformed the other two methods and produced smaller error during the test period for both ICH incidence (RMSE=1841 versus 6462 for Lee-Carter and 9498 for ARC) and mortality (RMSE=4033 versus 11199 for Lee-Carter and 13323 for ARC) (Figure S8).

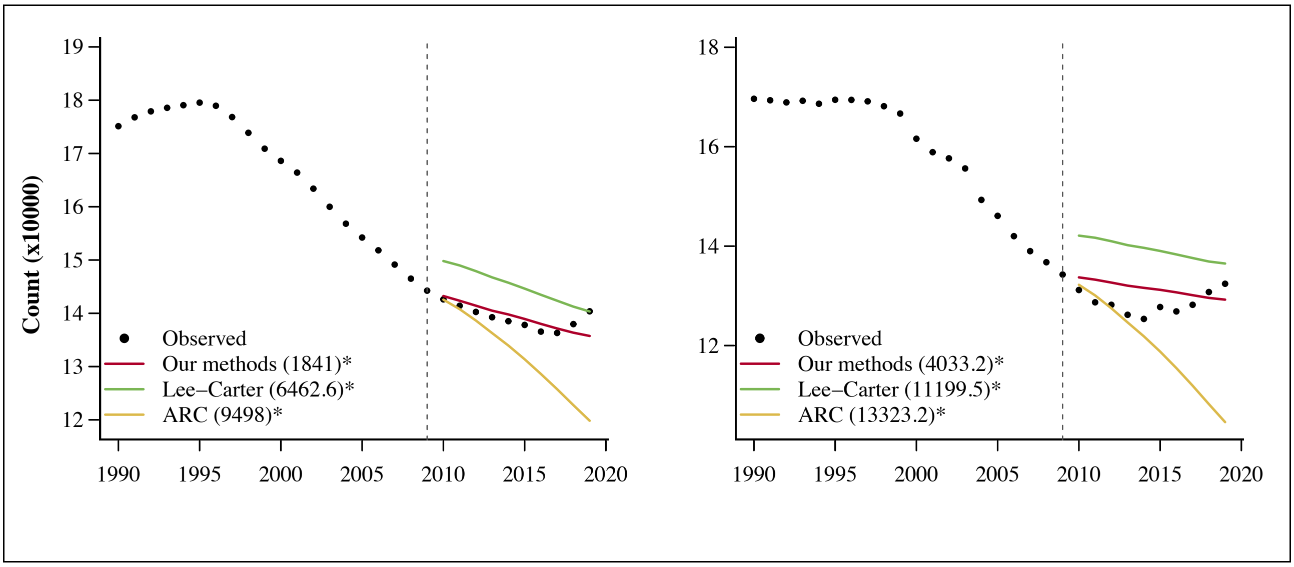

Figure S8: Overall out-of-sample predictions for ICH incidence and mortality in Europe.

*Numebers are the calculated root mean-squared error (RMSE) over the test period (2010-2019). ARC indicates average annualised rate of change method.

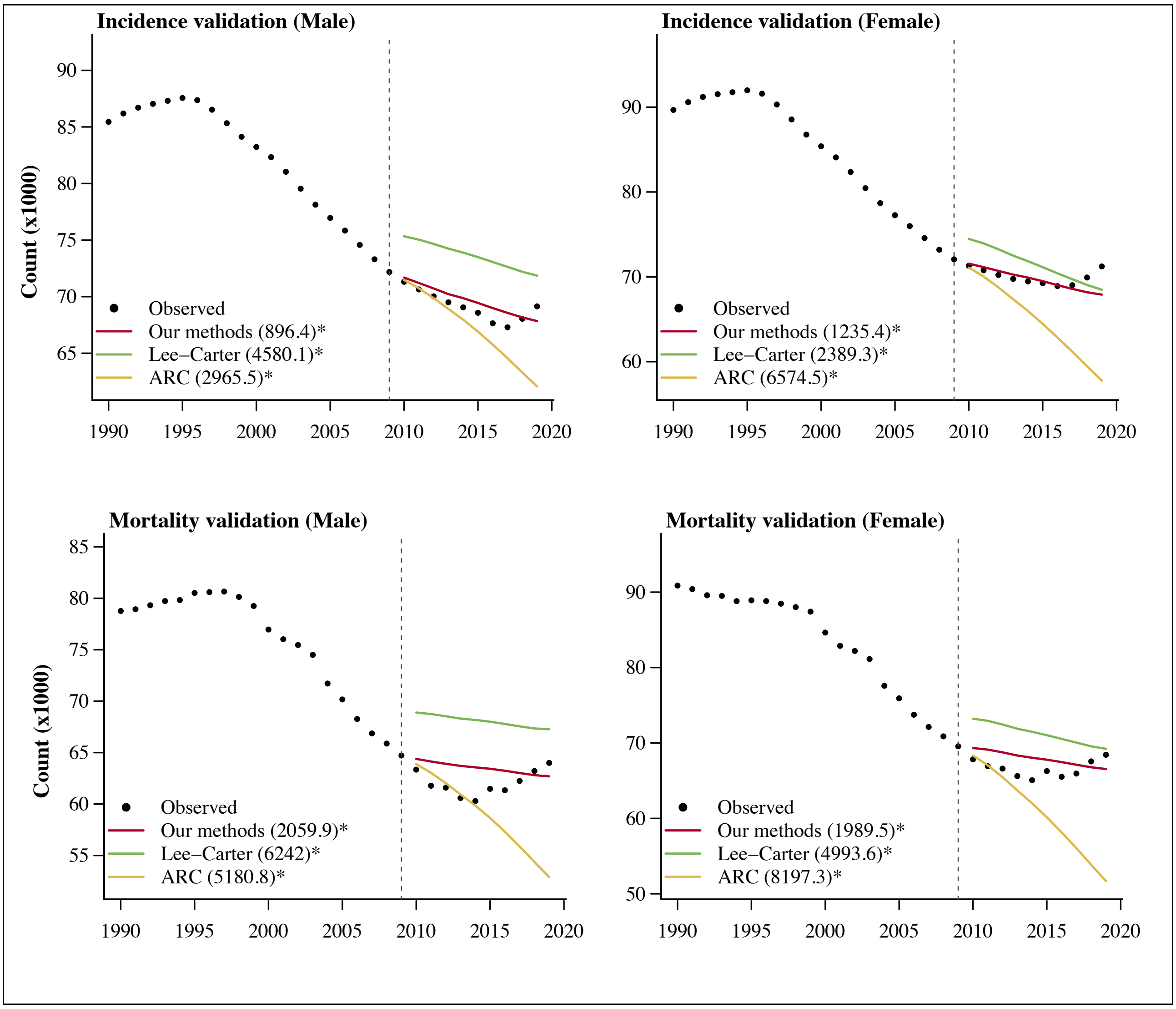

Figure S9: Out-of-sample predictions for ICH incidence and mortality in Europe by sex groups.

*Numebers are the calculated root mean-squared error (RMSE) over the test period (2010-2019). ARC indicates average annualised rate of change method.

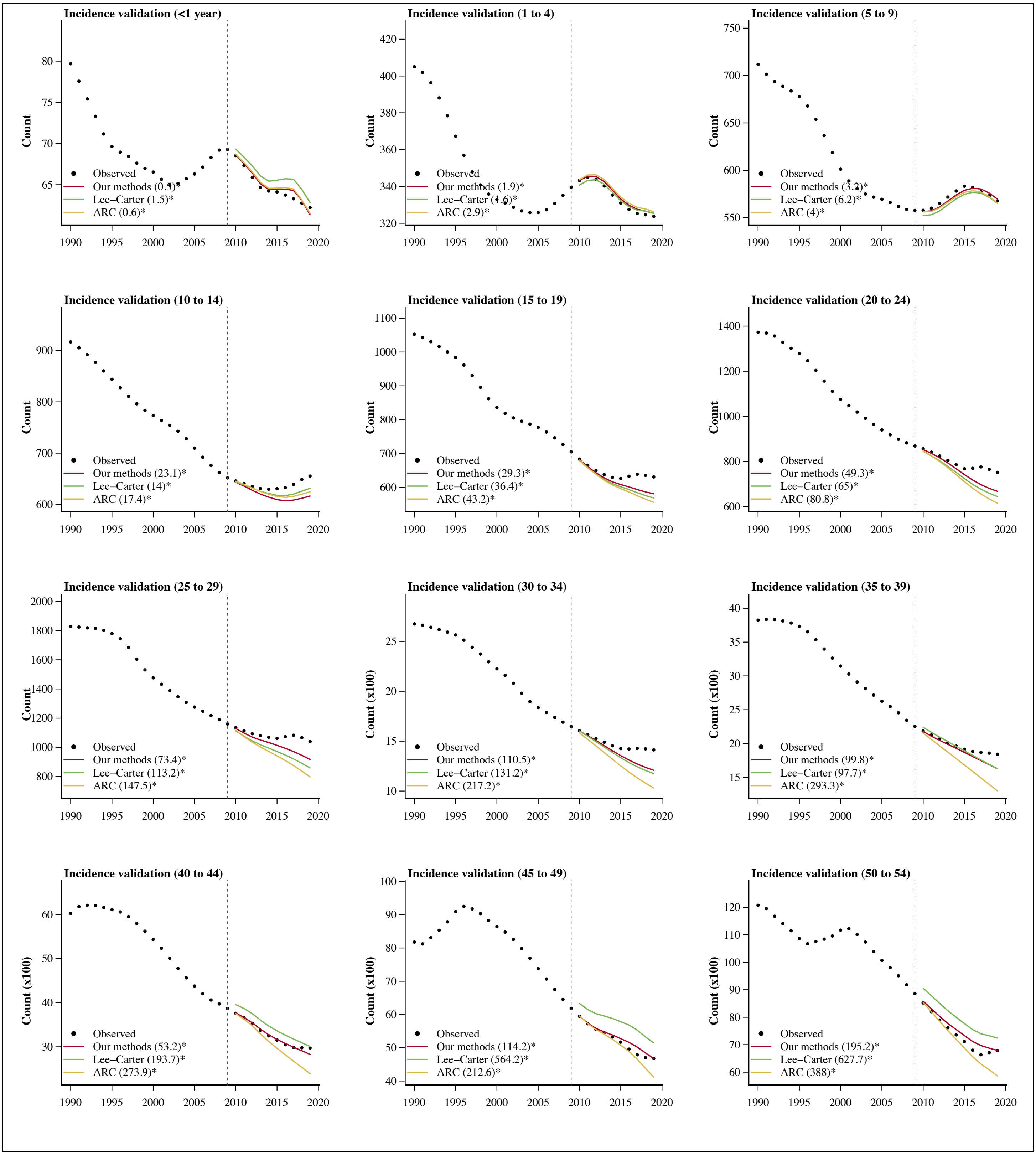
Incidence validation (<1 year)
Count
Observed
Our methods (0.5)*
Lee−Carter (1.5)*
ARC (0.6)*
Incidence validation (1 to 4)
Count
Observed
Our methods (1.9)*
Lee−Carter (1.6)*
ARC (2.9)*
Incidence validation (5 to 9)
Count
Observed
Our methods (3.2)*
Lee−Carter (6.2)*
ARC (4)*
Incidence validation (10 to 14)
Count
Observed
Our methods (23.1)*
Lee−Carter (14)*
ARC (17.4)*
Incidence validation (15 to 19)
Count
Observed
Our methods (29.3)*
Lee−Carter (36.4)*
ARC (43.2)*
Incidence validation (20 to 24)
Count
Observed
Our methods (49.3)*
Lee−Carter (65)*
ARC (80.8)*
Incidence validation (25 to 29)
Count
Observed
Our methods (73.4)*
Lee−Carter (113.2)*
ARC (147.5)*
Incidence validation (30 to 34)
Count (x100)
Observed
Our methods (110.5)*
Lee−Carter (131.2)*
ARC (217.2)*
Incidence validation (35 to 39)
Count (x100)
Observed
Our methods (99.8)*
Lee−Carter (97.7)*
ARC (293.3)*
Incidence validation (40 to 44)
Count (x100)
Observed
Our methods (53.2)*
Lee−Carter (193.7)*
ARC (273.9)*
Incidence validation (45 to 49)
Count (x100)
Observed
Our methods (114.2)*
Lee−Carter (564.2)*
ARC (212.6)*
Incidence validation (50 to 54)
Count (x100)
Observed
Our methods (195.2)*
Lee−Carter (627.7)*
ARC (388)*
1990 1995 2000 2005 2010 2015 2020

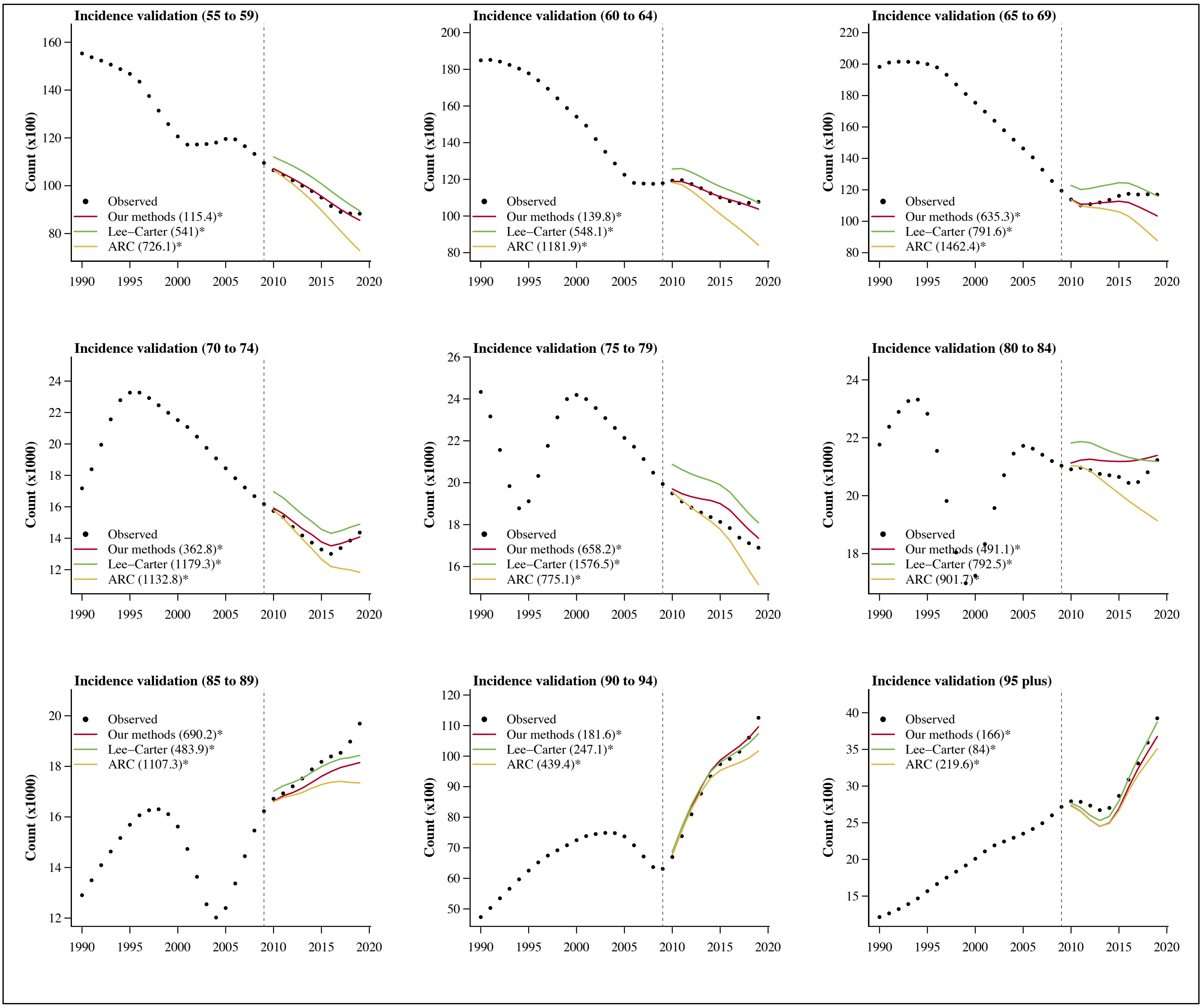

Figure S10: Out-of-sample predictions for ICH incidence in Europe by age groups.

*Numebers are the calculated root mean-squared error (RMSE) over the test period (2010-2019). ARC indicates average annualised rate of change method.

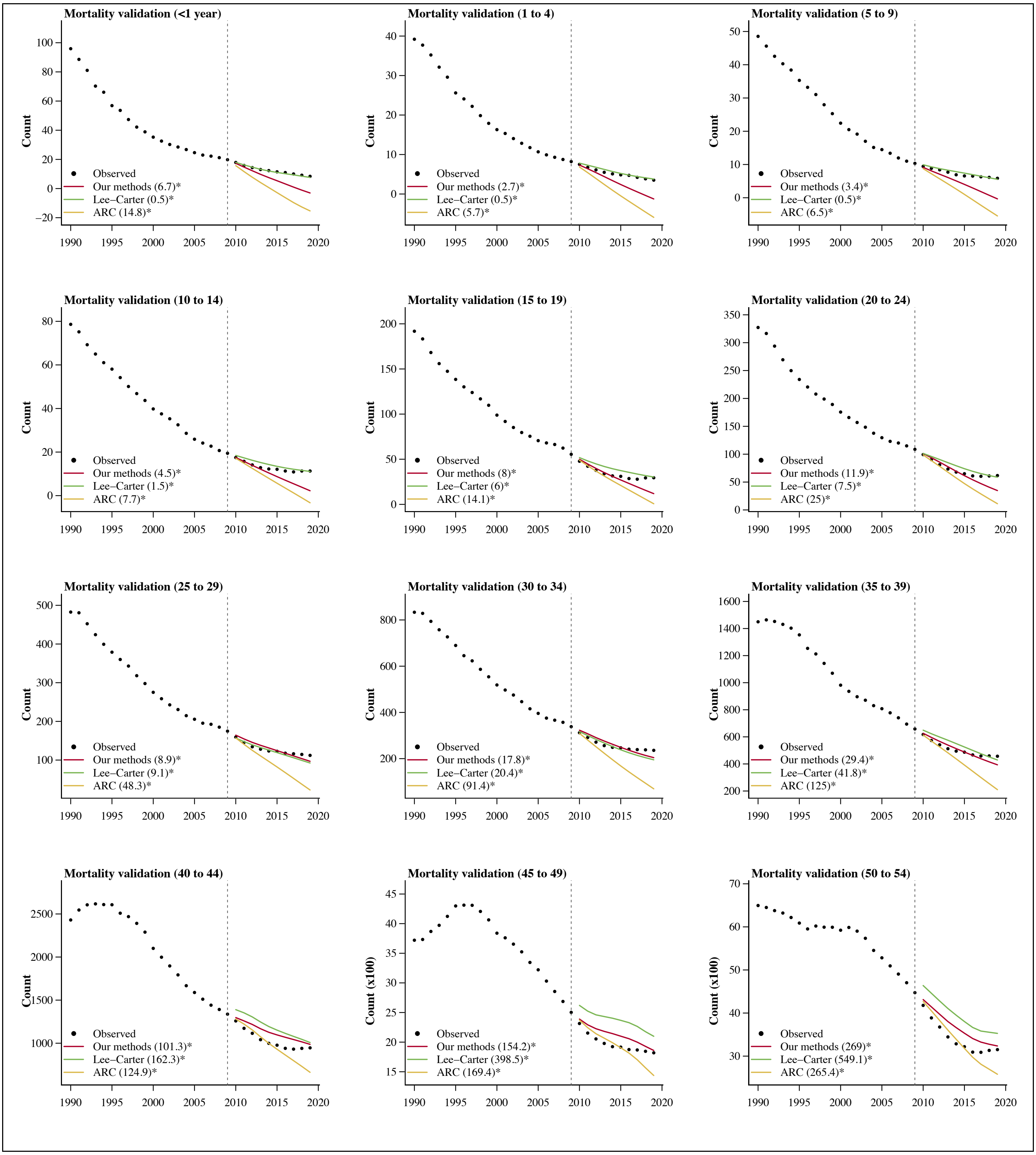

Mortality validation (<1 year)
Count
Observed
Our methods (6.7)*
Lee–Carter (0.5)*
ARC (14.8)*
Mortality validation (1 to 4)
Count
Observed
Our methods (2.7)*
Lee–Carter (0.5)*
ARC (5.7)*
Mortality validation (5 to 9)
Count
Observed
Our methods (3.4)*
Lee–Carter (0.5)*
ARC (6.5)*
Mortality validation (10 to 14)
Count
Observed
Our methods (4.5)*
Lee–Carter (1.5)*
ARC (7.7)*
Mortality validation (15 to 19)
Count
Observed
Our methods (8)*
Lee–Carter (6)*
ARC (14.1)*
Mortality validation (20 to 24)
Count
Observed
Our methods (11.9)*
Lee–Carter (7.5)*
ARC (25)*
Mortality validation (25 to 29)
Count
Observed
Our methods (8.9)*
Lee–Carter (9.1)*
ARC (48.3)*
Mortality validation (30 to 34)
Count
Observed
Our methods (17.8)*
Lee–Carter (20.4)*
ARC (91.4)*
Mortality validation (35 to 39)
Count
Observed
Our methods (29.4)*
Lee–Carter (41.8)*
ARC (125)*
Mortality validation (40 to 44)
Count
Observed
Our methods (101.3)*
Lee–Carter (162.3)*
ARC (124.9)*
Mortality validation (45 to 49)
Count (x100)
Observed
Our methods (154.2)*
Lee–Carter (398.5)*
ARC (169.4)*
Mortality validation (50 to 54)
Count (x100)
Observed
Our methods (269)*
Lee–Carter (549.1)*
ARC (265.4)*

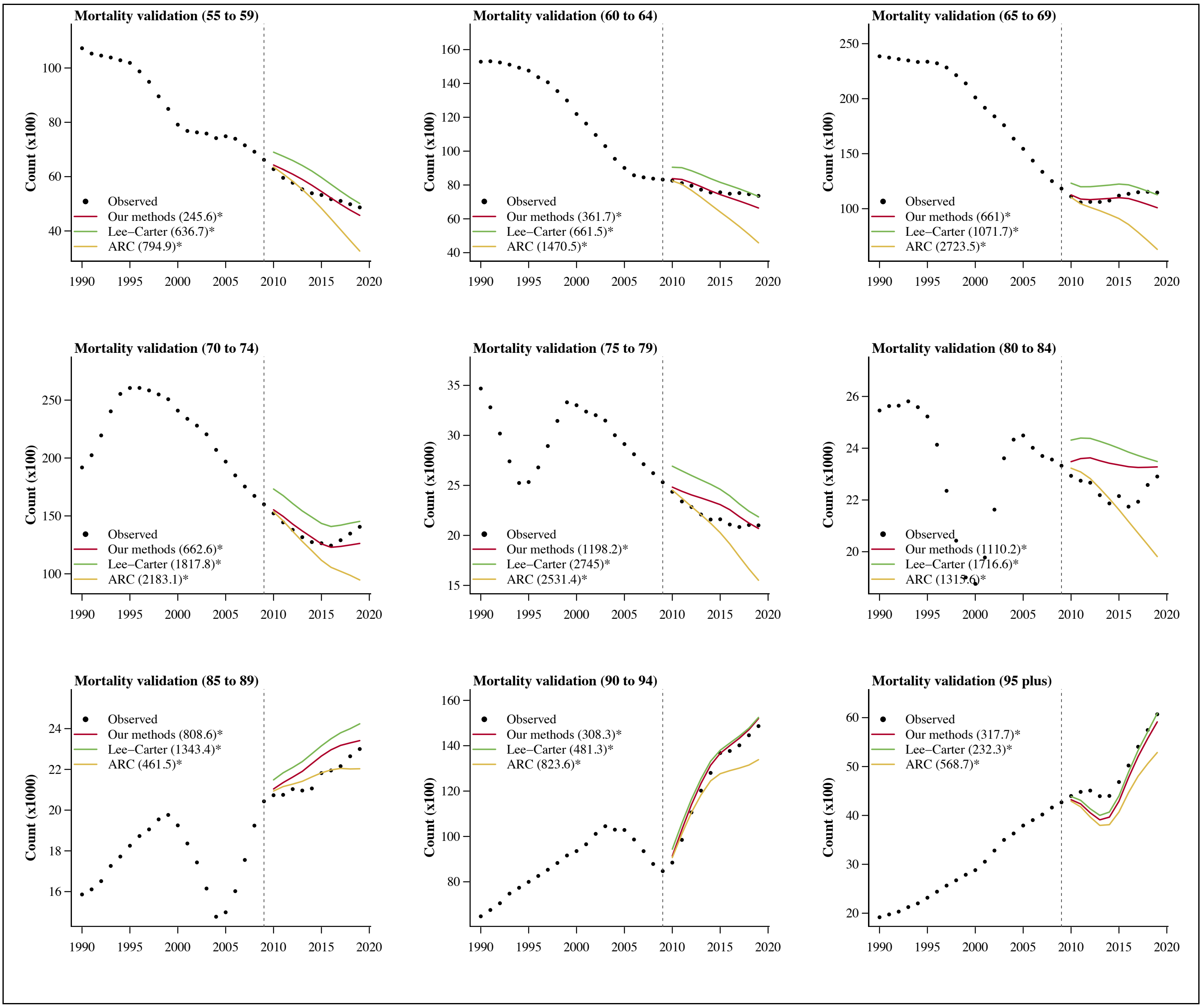

Figure S11: Out-of-sample predictions for ICH mortality in Europe by age groups.

*Numebers are the calculated root mean-squared error (RMSE) over the test period (2010-2019). ARC indicates average annualised rate of change method.

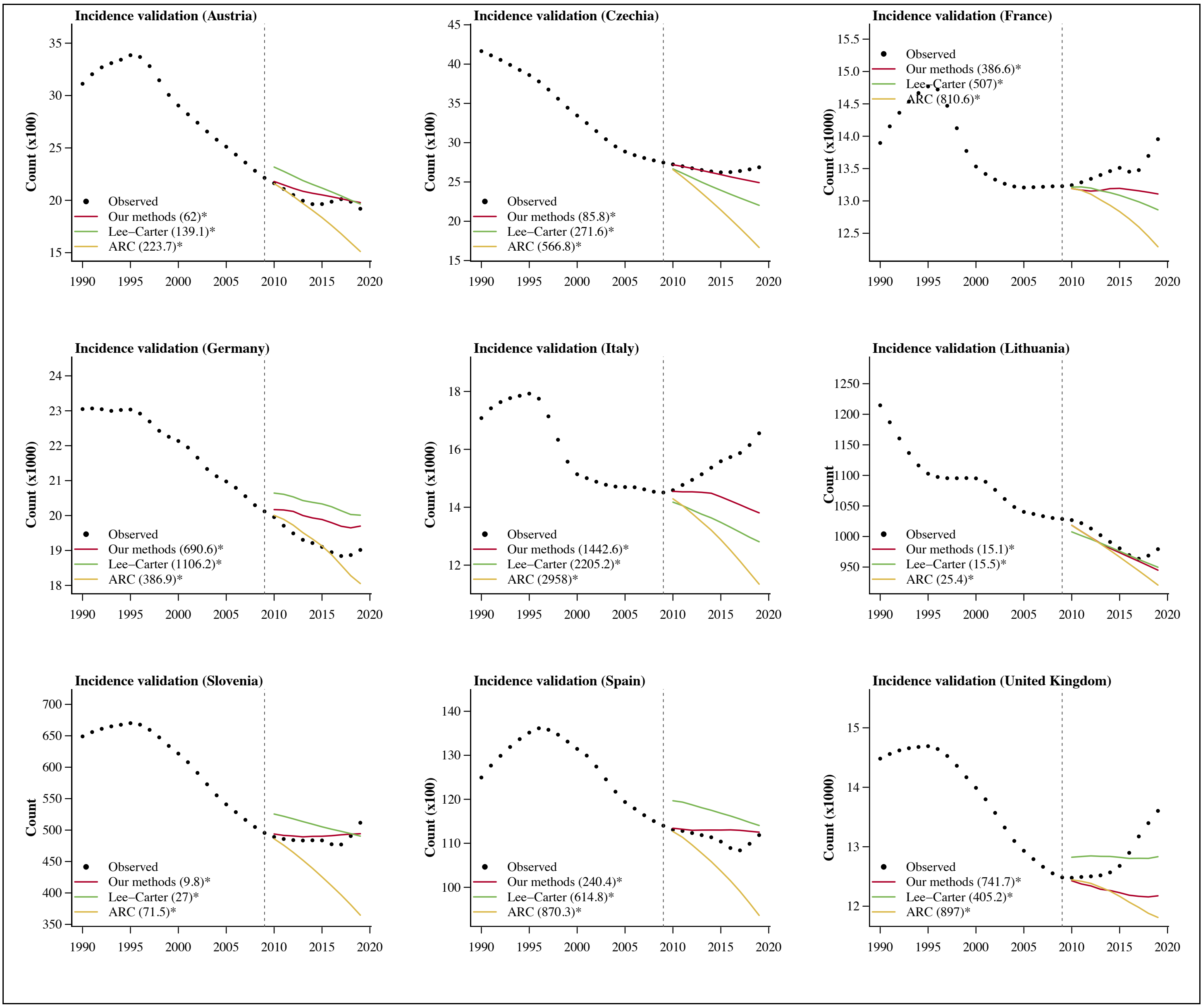

Figure S12: Out-of-sample predictions for ICH incidence in selected European countries.

*Numebers are the calculated root mean-squared error (RMSE) over the test period (2010-2019). ARC indicates average annualised rate of change method.

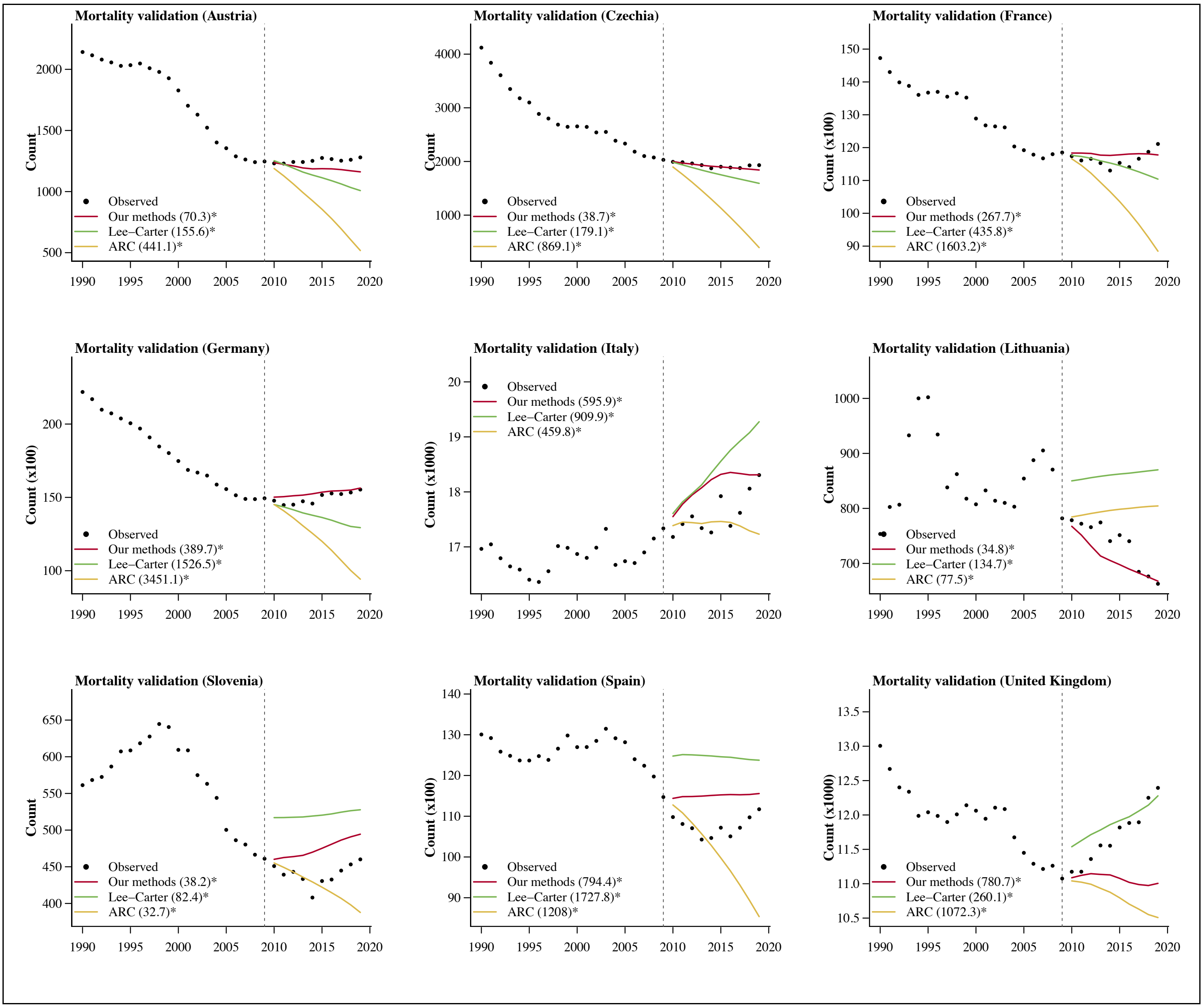

Figure S13: Out-of-sample predictions for ICH mortality in selected European countries.

*Numebers are the calculated root mean-squared error (RMSE) over the test period (2010-2019). ARC indicates average annualised rate of change method.

References

1. Feigin VL, Stark BA, Johnson CO, Roth GA, Bisignano C, Abady GG, et al. Global, regional, and national burden of stroke and its risk factors, 1990–2019: a systematic analysis for the Global Burden of Disease Study 2019. The Lancet Neurology. 2021 Oct 1;20(10):795–820.

2. Roth GA, Abate D, Abate KH, Abay SM, Abbafati C, Abbasi N, et al. Global, regional, and national age-sex-specific mortality for 282 causes of death in 195 countries and territories, 1980–2017: a systematic analysis for the Global Burden of Disease Study 2017. The Lancet. 2018;392(10159):1736–88.

3. Foreman KJ, Lozano R, Lopez AD, Murray CJ. Modeling causes of death: an integrated approach using CODEm. Population health metrics. 2012;10(1):1–23.

4. Stanaway JD, Afshin A, Gakidou E, Lim SS, Abate D, Abate KH, et al. Global, regional, and national comparative risk assessment of 84 behavioural, environmental and occupational, and metabolic risks or clusters of risks for 195 countries and territories, 1990–2017: a systematic analysis for the Global Burden of Disease Study 2017. The lancet. 2018;392(10159):1923–94.

5. Murray CJL, Aravkin AY, Zheng P, Abbafati C, Abbas KM, Abbasi-Kangevari M, et al. Global burden of 87 risk factors in 204 countries and territories, 1990–2019: a systematic analysis for the Global Burden of Disease Study 2019. The Lancet. 2020 Oct 17;396(10258):1223–49.

6. Aravkin A, Davis D. Trimmed statistical estimation via variance reduction. Mathematics of Operations Research. 2020;45(1):292–322.

7. Pavarin RM. Cocaine consumption and death risk: a follow-up study on 347 cocaine addicts in the metropolitan area of Bologna. Annali-Istituto Superiore di Sanita. 2008;44(1):91.

8. Foreman KJ, Marquez N, Dolgert A, Fukutaki K, Fullman N, McGaughey M, et al. Forecasting life expectancy, years of life lost, and all-cause and cause-specific mortality for 250 causes of death: reference and alternative scenarios for 2016–40 for 195 countries and territories. The Lancet. 2018 Nov 10;392(10159):2052–90.

9. O'Donnell MJ, Chin SL, Rangarajan S, Xavier D, Liu L, Zhang H, et al. Global and regional effects of potentially modifiable risk factors associated with acute stroke in 32 countries (INTERSTROKE): a case-control study. The lancet. 2016;388(10046):761–75.

10. Forouzanfar MH, Afshin A, Alexander LT, Anderson HR, Bhutta ZA, Biryukov S, et al. Global, regional, and national comparative risk assessment of 79 behavioural, environmental and occupational, and metabolic risks or clusters of risks, 1990–2015: a systematic analysis for the Global Burden of Disease Study 2015. The Lancet. 2016 Oct 8;388(10053):1659–724.

11. Vollset SE, Goren E, Yuan CW, Cao J, Smith AE, Hsiao T, et al. Fertility, mortality, migration, and population scenarios for 195 countries and territories from 2017 to 2100: a

forecasting analysis for the Global Burden of Disease Study. The Lancet. 2020;396(10258):1285–306.

12. Nichols E, Steinmetz JD, Vollset SE, Fukutaki K, Chalek J, Abd-Allah F, et al. Estimation of the global prevalence of dementia in 2019 and forecasted prevalence in 2050: an analysis for the Global Burden of Disease Study 2019. The Lancet Public Health. 2022 Feb 1;7(2):e105–25.
